# Improved Lignin Conversion to High-Value Aromatic Monomers through Phase Junction CdS with Coexposed Hexagonal (100) and Cubic (220) Facets

**DOI:** 10.1021/acsami.4c02315

**Published:** 2024-06-04

**Authors:** Zongyang Yue, Shibo Shao, Jialin Yu, Guanchu Lu, Wenjing Wei, Yi Huang, Kai Zhang, Ke Wang, Xianfeng Fan

**Affiliations:** † Institute for Materials and Processes, School of Engineering, The University of Edinburgh, Edinburgh EH9 3BF, U.K.; ‡ Petrochemical Research Institute, PetroChina Company Limited, Beijing 102206, China; § Beijing Key Laboratory of Emission Surveillance and Control for Thermal Power Generation, North China Electric Power University, Beijing 102206, China; ∥ Beijing Key Laboratory of Ionic Liquids Clean Process, CAS Key Laboratory of Green Process and Engineering, State Key Laboratory of Mesoscience and Engineering, Innovation Academy for Green Manufacture, Institute of Process Engineering, Chinese Academy of Sciences, Beijing 100190, China; ⊥ Longzihu New Energy Laboratory, Zhengzhou Institute of Emerging Industrial Technology, Henan University, Zhengzhou 450000, China

**Keywords:** phase junction cadmium sulfide nanoparticles, coexposed hexagonal (100) and cubic (220) facets, photocatalysis, C_β_−O bonds’ cleavage, lignin valorization

## Abstract

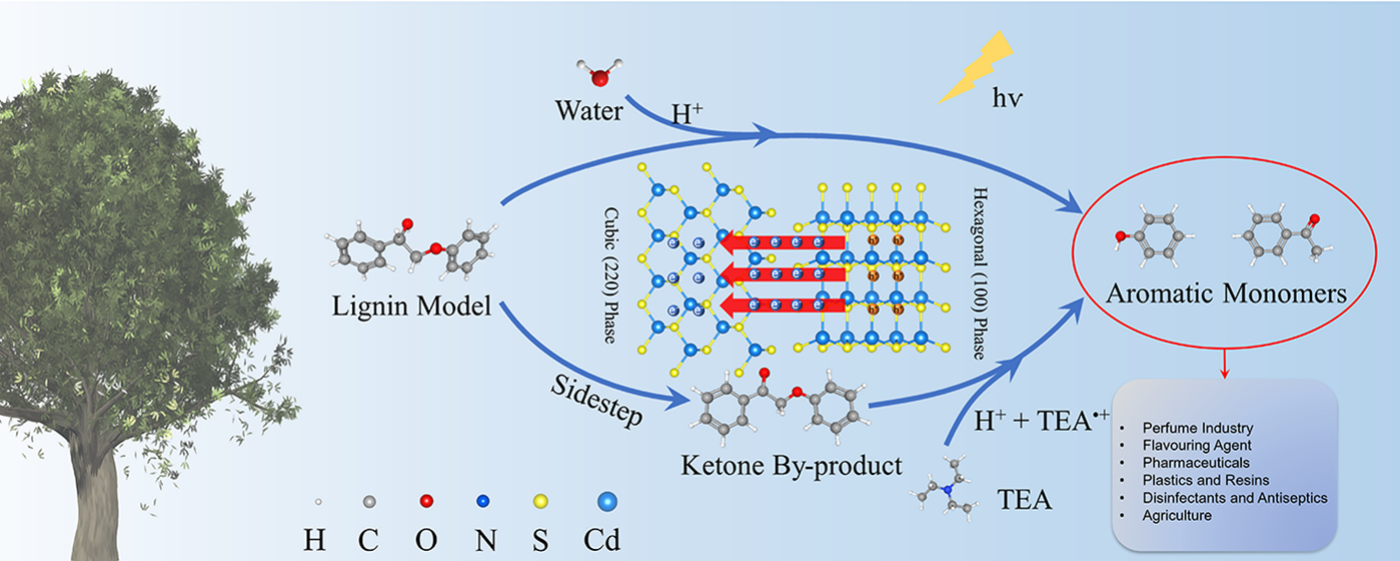

Photocatalysis has the potential for lignin valorization to generate functionalized aromatic monomers, but its application has been limited by the slow conversion rate and the low selectivity to desirable aromatic products. In this work, we designed the phase junction CdS with coexposed hexagonal (100) and cubic (220) facets to improve the photogenerated charge carriers’ transfer efficiency from (100) facet to (220) facet and the hydrogen transfer efficiency for an enhanced conversion rate of lignin to aromatic monomers. Water is found as a sufficient external hydrogen supplier to increase the yields of aromatic monomers. These innovative designs in the reaction system promoted complete conversion of PP-ol to around 94% of aromatic monomers after 1 h of visible light irradiation, which shows the highest reaction rate and selectivity of target products in comparison with previous works. PP-one is a byproduct from the overoxidation of PP-ol and is usually difficult to be further cleaved to acetophenone and phenol as the desirable aromatic monomers. TEA was first identified in this study as a sacrificial electron donor, a hydrogen source, and a mediator to enhance the cleavage of the C_β_–O bonds in PP-one. With the assistance of TEA, PP-one can be completely cleaved to desirable aromatic monomer products, and the reaction time is reduced from several hours to 10 min of visible light irradiation.

## 1 Introduction

Lignin is one of the main components of lignocellulose.^1−3^ Due to its structural recalcitrance and complexity, lignin has not been effectively utilized and is mainly used to produce low-value products, such as activated carbon and fertilizers, or simply combusted for power generation.^4,5^ Efficient conversion of lignin into value-added products, such as aromatic monomers, can promote the utilization of these abundant renewable biomass resources.^4,5^ The aromatic-structured units in lignin are generally connected by C–C bonds and C–O bonds and more than 50% of aromatic groups are connected by C_β_–O bonds.^4,6,7^ The selective cleavage of interunit C_β_–O bonds between the aromatic units in lignin has been considered a critical stage in the conversion of lignin to high-value products. However, the traditional thermo-catalytic methods developed for this purpose generally require harsh reaction conditions, causing high-energy consumption.^8−10^ The reaction selectivity is also low, producing a high portion of low-functionalized aromatics, such as cyclohexanol and benzene, and even the formation of dark substances, known as humins.

Photocatalysis has a high potential for lignin conversion with a high selectivity of high-functionalized aromatic monomers and a low energy consumption under mild reaction conditions in comparison to traditional thermos-catalysis.^11,12^ In the photocatalytic lignin conversion process, the formation of C_α_ radical intermediates via C_α_–H bonds activation is an important step due to the remarkable decrease in the bond dissociation energy of C_β_–O bonds from 55 kcal/mol in PP-ol to 7.8 kcal/mol, therefore facilitating the cleavage of C_β_–O bonds in lignin to aromatic monomers.^4^ Moreover, the hydrogen transfer efficiency during cleavage of the C_β_–O bonds is also critical to the selectivity of the target monomeric products. The high hydrogen transfer efficiency can facilitate the hydrogen migration from the surface of photocatalysts to the aromatic monomeric radicals, thereby improving the generation of desirable aromatic monomers. For example, in the reaction system with a low hydrogen transfer efficiency, DB-one was produced as the C–C coupling byproduct from the acetophenone radical, rather than acetophenone as the target aromatic monomers, and therefore decreased the selectivity of high-value aromatic monomers.^7^ In previous research, various rational designs of photocatalysts, such as optimizing energy band structure, adjusting Fermi level, boosting visible light adsorption capability, and improving close contact between photocatalysts and lignin, have been proposed to improve the reaction rate and selectivity to aromatic monomeric products.^13−16^ For example, Wang’s group indicated that the optimization of energy band structure in zinc–indium-sulfide photocatalysts by adjusting the atomic ratio of Zn/In can improve the photocatalytic performance.^14^ Yoo et al. developed the Ag^+^ exchanged CdS nanoparticles to adjust the Fermi level and thus provide more photogenerated electrons and holes for the cleavage of C_β_–O bonds reaction.^13^ Li’s group designed a CdS–SH/TiO_2_ heterojunction photocatalyst in which the -SH groups can provide close-contact between the photocatalyst and lignin to effectively improve the cleavage of C_β_–O bonds in lignin.^17^ Yao et al. prepared ZIF-8-NH_2_@Bi/Bi_2_MoO_6_ with oxygen vacancies to improve visible light harvesting capability through the SPR effect in the Bi nanoparticles on the photocatalysts to enhance the conversion rate to aromatic monomers.^18^ Their research promoted significant improvement in the conversion of lignin to aromatic monomers, but the reaction rate and selectivity still have some space to improve. Phase junction CdS photocatalysts have been used for effective hydrogen evolution, because of its improved charged carriers’ separation efficiency, and tunable energy band structure.^19−23^ We may use similar approaches to turn the energy band structure and improve the charge carriers’ separation efficiency to improve the activation of C_α_–H bonds and cleavage of the C_β_–O bonds in lignin. In addition, hydrogen transfer efficiency is another important factor controlling the yields of aromatic monomers. The facilitating hydrogen transfer efficiency can avoid the cleavage of C_β_–O bonds to form DB-one byproduct. To solve these problems, we designed the coexposed cubic (220) and hexagonal (100) facets on the phase junction CdS nanoparticles with the excellent photogenerated charge carriers’ migration efficiency to improve the activation of C_α_–H bonds and the facilitated hydrogen transfer efficiency to decrease the yields of DB-one byproduct that can significantly improve the conversion rate to desirable aromatic monomers.

The selectivity to produce high-value aromatic monomers from lignin model is limited by the production of ketone compound, which is a major byproduct and needs to be further converted to desirable aromatic monomers. The effective conversion of a ketone compound to desirable aromatic monomers is an essential step for the scaling up of photocatalytic lignin conversion. In literature, the fragmentation of ketone compound is relatively low and is mainly controlled by both the external hydrogen supply and the activation of C_α_=O bonds in ketone compound.^6,13^ To date, the time to achieve 100% of conversion of ketone compound is from 5 to 12 h of visible light irradiation.^6,24,25^ In addition, simply increasing external hydrogen donors, for example, adding water, isopropyl alcohol, or ethanol, the hydrogenolysis of ketone byproduct still could not be efficiently performed.^26^ The new mechanism of C_α_=O bonds’ activation and reducing the energy barrier to promote the cleavage of the C_β_–O bonds in ketone compound needs to be developed for achieving high production of desirable aromatic monomeric products. In this study, the conversion of ketone byproduct into aromatic monomers is facilitated through reducing the energy barrier for the activation of C_α_=O bonds in ketone compound with the assistance of triethylamine (TEA), in which TEA acts as a sacrificial electron donor, proton source, and mediator.

## 2 Experimental Methods

### 2.1 Materials

Trisodium citrate dihydrate (99%+), thioacetamide (99%+), acetonitrile (CH_3_CN), and 1,2-dibenzoyl-1,4-butanedione (DB-one) were purchased from Fisher Scientific International, Inc. 2-Phenoxy-1-phenylethanol (PP-ol) and 2-phenoxyacetophenone (PP-one) were purchased from Fluorochem Ltd. 5,5-Dimethyl-1-pyrroline N-oxide (DMPO), Cd(NO_3_)_2_·4H_2_O (98%), phenol, acetophenone, sodium sulfide nonahydrate (98%+), sodium persulfate (99%), sodium sulphite (98.5%), D-mannitol, (3-Bromopropyl) trimethoxysilane (BPTMOS), TEA and Sodium hydrosulfide hydrate were purchased from Sigma-Aldrich Co., Ltd. 2-(2-Methoxyphenoxy)-1-phenylethanol (MP-ol), 2-phenoxy-1-phenylpropane-1,3-diol (PPP-ol), 1-(3,4-dimethoxyphenyl)-2-(2-methoxyphenoxy)propane-1,3-diol (DMP-ol), and Phenethoxybenzene (PEB) was purchased from BLD Pharmatech Ltd. Ethylene glycol (EG) was purchased from VWR International, LLC.

### 2.2 Preparation of Phase Junction CdS with Coexposed Hexagonal (100) and Cubic (220) Facets

The phase junction CdS was prepared by a one-pot hydrothermal method. Typically, 308 mg Cd(NO_3_)_2_·4H_2_O and a certain amount (*n* mg, *n* = 50, 100, 150, 200, 300) of trisodium citrate were dissolved into 15 mL of water and EG mixed solution (v_water_/v_EG_ = 1/5). After being ultrasonically dispersed for 10 min and vigorously stirred for 30 min, 375 mg of thioacetamide was added to the solution. After the mixture was stirred for another 30 min, it was transferred into a 25 mL stainless Teflon-lined autoclave reactor. The autoclave reactor was subsequently heated to 160 °C with a 3 °C min^–1^ of heating rate in an oven and kept the temperature for 4 h. After natural cooling, the sample was collected by centrifugation (9000 rpm) and rinsed several times with ethanol and water, respectively. The solid samples were then dried under a vacuum at 60 °C for 4 h. The obtained catalyst was labeled as CdS-*n*: CdS-50, CdS-100, CdS-150, CdS-200 and CdS-300, where *n* is the amount of trisodium citrate added in the solution. The pristine CdS (CdS-0) was also synthesized via the same process but without trisodium citrate.

### 2.3 Photocatalytic Activity Evaluation

Photocatalytic reactions were performed in a customized quartz reactor with a cooling water jacket. The visible light source was provided by a xenon arc lamp (Perfect Light Company) equipped with a 420 nm UV filter. The position of the lamp was fixed during the entire experiment to maintain the constant light intensity of 0.35 W cm^–2^. Initially, 10 mg of reactants (PP-ol, MP-ol, or PP-one), and 10 mg of photocatalyst were dispersed in 5 mL of solvent (CH_3_CN, CH_3_CN/H_2_O mixture, ethanol, methanol, or isopropanol) in the reactor. The cooling water was employed to keep the temperature of reactor at 20 °C. The photocatalysts were dispersed by magnetic stirring, and the reactor was firmly sealed after 30 min of argon purge (10 mL min^–1^). The sealed reactor with 200 rpm of magnetic stirring was illuminated under visible light irradiation. After the reaction, the solution was collected by centrifugation, and methylparaben was added as the internal standard. The solution was qualitatively analyzed by the gas chromatography–mass spectrometry (GC–MS, Rxi-5 ms column, QP2010 SE, Shimadzu) and quantitatively analyzed by the gas chromatography (GC, Stabilwax-MS column, GC-2010 plus, Shimadzu). The following equations were used to calculate the conversion rate of reactants, the selectivity of products and the yields of products respectively:
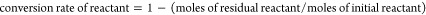
1
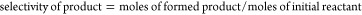
2
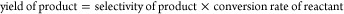
3

### 2.4 Alkylation and Regeneration Experiments

The alkylation and regeneration of sulfur moieties experiments were modified from the method reported in the literature.^14^ For the inhibition of sulfur moieties on the surface of CdS-150, 50 mg of CdS-150 and 20 μL of BPTMOS were added into 10 mL of cyclohexane and then stirred at 80 °C for 6 h. After the reaction, BPTMOS treated CdS-150 was collected by centrifugation and rinsed with cyclohexane three times before being dried under vacuum at 60 °C for 4 h. As for the regeneration, the inhibition of sulfur moieties on the surface could be removed by an aqueous solution of NaSH. Specifically, 30 mg of BPTMOS treated CdS-150 photocatalyst and 100 mg of NaSH were added into 10 mL of water, and it was then stirred at 60 °C for 2 h. After regeneration, the obtained CdS-150 was collected by centrifugation. The sample was washed with water three times and then dried in a vacuum oven at 60 °C for 4 h.

### 2.5 Characterization

The scanning electron microscopy (SEM, Zeiss Sigma VP) and high-resolution transmission electron microscopy (TEM/HRTEM, JEOL JEM-2100F) were employed to image the morphologies of as-prepared CdS. X-ray diffraction patterns (XRD) were acquired with a Bruker Phaser-D2 diffractometer. The BET results of CdS were conducted with a Quantachrome IQ sorption analyzer. UV–vis diffuse reflectance spectroscopy (DRS) was performed using a JASCO V-670 spectrophotometer. X-ray photoelectron valence band spectra (XPS-VB) were conducted by an X-ray photoelectron spectrometer (ThermoFisher K-Alpha) to analyze the electronic configuration and chemical states of each element in CdS photocatalysts. The Raman spectra of CdS were recorded on a Hrobia Xplra plus Raman with a 532 nm solid laser as the exciting source. Photoluminescence (PL) analysis was conducted on a Shimadzu RF-6000. The time-resolved photoluminescence (TRPL) experiment was conducted on an Edinburgh FLS1000. The contact angle of CdS was measured on a First Ten Angstrom (FTA200, Portsmouth, VA) video system with FTA32 software. The photoelectrochemical (PEC) measurements were performed in a standard three-electrode system by using an electrochemical workstation (CHI660E, Chenhua, shanghai) under visible light illumination in 15 mL of CH_3_CN and 35 mL of H_2_O with 30 mg of PP-ol and 0.2 M Na_2_ClO_4_.

## 3 Results and Discussion

### 3.1 Characterization of Photocatalysts

**Figure 1:**
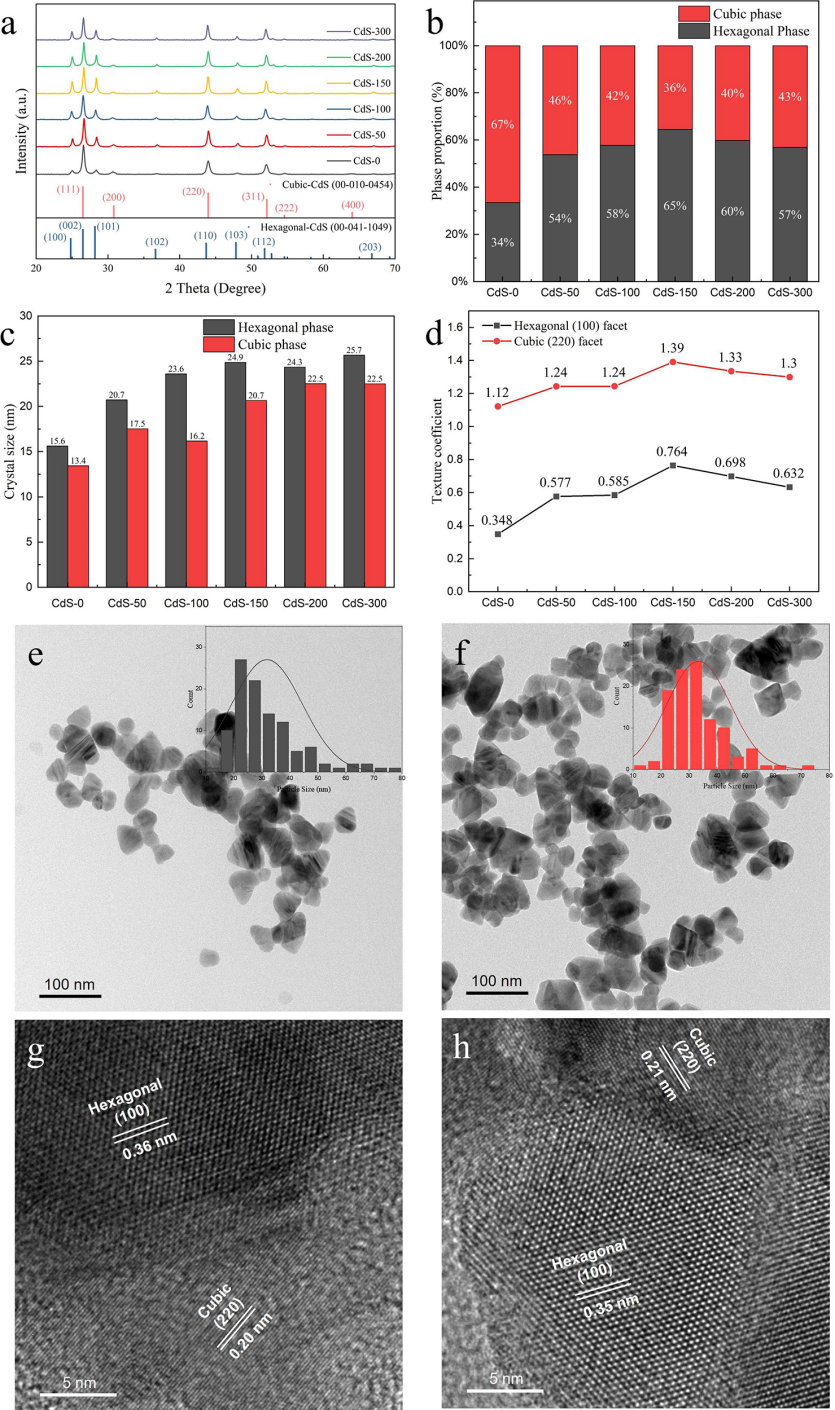
(a) XRD patterns of CdS-*n*; (b) The ratio of hexagonal and cubic in CdS-*n* photocatalysts. (c) The crystal sizes of both the hexagonal and cubic phases in CdS-*n* photocatalysts through the Scherrer equation. (d) The texture coefficient of the hexagonal (100) facet and cubic (220) facet in CdS-*n* photocatalysts. TEM and HRTEM images of CdS-0 (e, g) and CdS-150 (f, h). The inset graphs in (e) and (f) are corresponding particle size distributions.

The XRD, Raman, XPS, SEM and TEM characterizations demonstrate that the phase junction CdS with coexposed cubic (220) and hexagonal (100) facets were successfully synthesized through controlling the ratio of Cd^2+^/S^2–^ during the in situ crystal formation process via the chelating effect caused by the added trisodium citrate surfactant. XRD patterns demonstrate that phase junction CdS-*n* photocatalysts predominantly exhibit coexposed cubic (220) and hexagonal (100) facets. Figure 1a presents the XRD results of the as-prepared CdS-*n* photocatalysts and the standard patterns of CdS (hexagonal phase of CdS: JCPDS No. 41-1049; cubic phase of CdS: JCPDS No. 10-0454). These results indicate that all as-prepared CdS photocatalysts contain both hexagonal and cubic phases. Based on XRD results, the ratio of hexagonal to cubic phases in phase junction CdS-*n* photocatalysts was obtained and is presented in Figure 1b. These results indicate that the ratio of hexagonal phase to cubic phase varies with the applied dose of trisodium citrate and reach the highest value when 150 mg of trisodium citrate (CdS-150) is used. The crystal sizes for the hexagonal and cubic phases within the CdS-*n* photocatalysts were calculated through Scherrer equation and the detailed calculations were presented in Supporting Information.^27−29^ As shown in Figure 1c, the crystal sizes for each phase in CdS photocatalysts gradually increase with the dose of trisodium citrate used in the synthesis of CdS-0 to CdS-300. The increase in the crystal size potentially improves the exposure of the cubic (220) and hexagonal (100) facets on the CdS photocatalysts. The texture coefficients of cubic (220) and hexagonal (100) facets were calculated and presented in Figure 1d to further investigate the amount of coexposed cubic (220) and hexagonal (100) facets in phase junction CdS-*n* photocatalysts. The results demonstrate that the amount of cubic (220) and hexagonal (100) facets varies with the applied dose of trisodium citrate and reaches the highest amount when 150 mg of trisodium citrate (CdS-150) is used.

TEM and SEM images further presented a close interconnection between cubic (220) and hexagonal (100) facets on phase junction CdS-*n* photocatalysts. As shown by the TEM and SEM results in Figures 1e-f and S1, both CdS-0 and CdS-150 are irregular nanoparticles, and the particle size slightly increases from ∼25 nm in CdS-0 to ∼30 nm in CdS-150. The HRTEM (Figures 1g-h) images show that both CdS-0 and CdS-150 have two representative crystal phases: hexagonal phase (100) and cubic phase (220). These two facets are interconnected to create a coexposed hexagonal (100) facet and cubic (220) facet.

**Figure 2:**
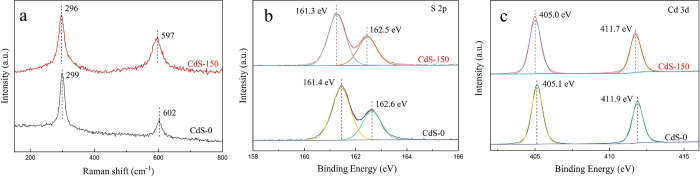
(a) Raman spectra of CdS-0 and CdS-150; High-resolution XPS spectra of CdS-0 and CdS-150: (b) S 2p states, (c) Cd 3d states.

Raman spectroscopy was employed to further analyze the structural properties and the crystallinity of phase junction CdS catalysts.^30^ In Figure 2a, the Raman spectrum of CdS-0 exhibits two types of scattering at 299 and 602 cm^–1^, which represents the cubic phase (1LO) and hexagonal phase (1LO and 2LO), respectively.^21,31^ The Raman peaks of CdS-150 are broader and blue-shifted in comparison to CdS-0, and the 2LO peak of CdS-150 is significantly higher, which strongly confirms that the dual-phase coexist in both samples and the portion of the hexagonal phase in CdS-150 is higher than CdS-0.^21,30−32^

XPS was used to analyze the electronic configuration and chemical states of CdS-0 and CdS-150. As shown in Figure S2, the full XPS spectrum demonstrates cadmium (Cd) and sulfur (S) as the predominant elements. The two peaks corresponding to the S 2p in CdS-0 (Figure 2b) are located at 161.4 eV (S 2p_3/2_) and 162.6 eV (S 2p_1/2_), but the S 2p peaks in CdS-150 are shifted to 161.3 and 162.5 eV, respectively. The high-resolution XPS of Cd 3d (Figure 2c) exhibits two peaks at 405.1 and 411.9 eV for CdS-0, which correspond to Cd 3d_5/2_ and Cd 3d_3/2_ respectively. Compared with CdS-0, the high-resolution XPS results of Cd 3d for CdS-150 show a slightly negative shift to the lower bonding energies. The shift of S 2p peaks and Cd 3d peaks reveals that the electron density in CdS-150 is higher than that in CdS-0.^33^

### 3.2 Improve Charge Carriers’ Transfer Efficiency and Visible Light Adsorption Capability through Coexposed (100) and (220) Facets

**Figure 3:**
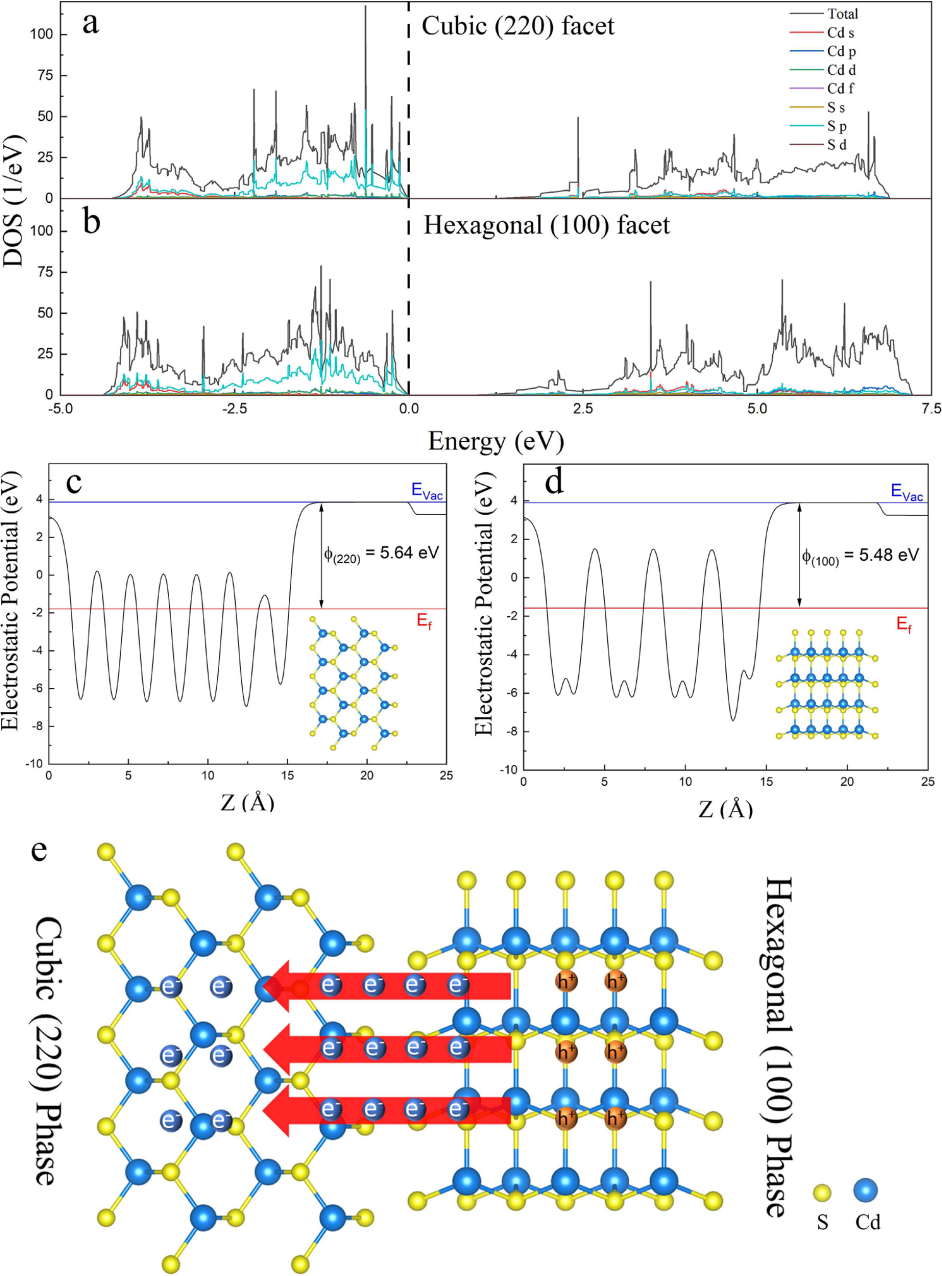
DOS of (a) cubic (220) facet and (b) hexagonal (100) facet. Calculated average potential profile along the Z axis of (c) cubic (220) facet and (d) hexagonal (100) facet. (e) The schematic photogenerated electrons transfer direction.

The DFT calculation, PEC, PL, TRPL and DRS results demonstrate that the coexposed hexagonal (100) and cubic (220) facets in phase junction CdS nanoparticles can facilitate the photocharge carriers’ transfer from hexagonal (100) facet to cubic (220) facet, improve the visible light absorption capability, and adjust the photoredox capability. Based on the DFT results, it is plausible that the charge carriers transfer from hexagonal (100) facets to cubic (220) facets, where hexagonal (100) facets act as electron providers, and cubic (220) facets accept photoexcited electrons. As shown in Figures 3a-b, both facets show similar DOS structures. The valence band maximum (VBM) of hexagonal (100) facet and cubic (220) facet is mainly contributed by the p orbit of S, while the conduction band maximum (CBM) of two facets is determined by the d orbit of Cd and the p orbit of S. It has been reported that the photogenerated charge carriers’ transfer efficiency could be improved by the construction of intimate interfaces and elimination of interfacial potential between the two facets in phase junction photocatalysts with similar crystal structures.^20^ In our study, the hexagonal and cubic phases show the similar DOS structure and crystal structure, which can facilitate the interatomic s-p and p-d hybridizations to form smooth bridges and optimize the photogenerated charge carriers’ transfer between hexagonal (100) facet and cubic (220) facet. Subsequently, the work functions of the two facets were calculated to determine the charge carriers’ transfer direction. As shown in Figures 3c-d, the energy of work function can be obtained through the vacuum level and Fermi level of hexagonal (100) facet and cubic (220) facets, corresponding to 5.48 eV in hexagonal (100) facet and 5.64 eV in cubic (220) facet. The difference in the energy of work function can lead the charge redistribution on the interface of both facets,^20^ and promote the photogenerated electrons transfer from hexagonal (100) facet to cubic (220) facet (Figure 3e). In this process, the hexagonal (100) facet and the cubic (220) facet tend to accumulate photogenerated holes and electrons, respectively.

The PEC results indicate that the increased amount of coexposed hexagonal (100) and cubic (220) facets in phase junction CdS photocatalysts improves the photogenerated electrons transfer from the (100) facet to (220) facet and reduces the charge carriers’ transfer resistance. The on/off transient photocurrent response and electrochemical impedance spectroscopy (EIS) in PEC characterizations were conducted to investigate both of the above-mentioned photophysical properties of CdS photocatalysts. As shown in Figure S3a, CdS-150 photocatalysts exhibit the highest density of photocurrent than other photocatalysts. The highest amount of coexposed hexagonal (100) and cubic (220) facets in CdS-150 photocatalysts improves the separation efficiency of charge carriers. The EIS results further demonstrate that CdS-150 has smaller arc radius (Figure S3b), indicating that the charge transfer resistance of CdS-150 is lower than that of other samples. Both on/off transient photocurrent response and EIS results demonstrate that the phase junction CdS-150 photocatalysts with the high amount of coexposed (100) and (220) facets can improve the separation efficiency of photogenerated charge carriers and decrease the charge carriers’ transfer resistance, therefore enhancing the photocatalytic performance in lignin conversion using CdS-150.

PL spectra and TRPL spectra were measured to investigate the effect of amount of coexposed (100) and (220) facets in phase junction CdS photocatalysts on the recombination time of photogenerated charge carriers and migration dynamics. The PL excitation spectra of CdS-0 and CdS-150 (Figure S4a) were measured under the emission wavelength of 595 nm to obtain the optimal excitation wavelength at 400 nm for both photocatalysts. Hence, the emission spectra of both photocatalysts were measured at 400 nm excitation wavelength to investigate the quenched efficiency of charge carriers. Generally, the higher quenched efficiency of photocatalysts represents the longer recombination time of photogenerated electrons and holes. As shown in Figure S4b, the emission intensity of CdS-150 is lower than that of CdS-0, indicating that CdS-150 shows the higher quenched efficiency and suppresses the recombination time of photogenerated electrons and holes. These results are mainly attributed to the fact that CdS-150 with the highest amount of coexposed (100) and (220) facets can effectively transfer the photogenerated electrons from the hexagonal (100) facet to the cubic (220) facet, therefore prolonging the recombination time. To further investigate their charge carriers decay lifetime, TRPL analyses of both samples were conducted and presented in Figure S4c. The results indicate that the average emission lifetime (τ_ave_) for the two samples follows a second-order kinetic process. In detail, CdS-0 exhibits τ_1_ and τ_2_ of 0.38 and 2.72 ns, respectively, while CdS-150 shows τ_1_ and τ_2_ of 0.39 and 5.21 ns, respectively. The τ_ave_ was calculated based on τ_ave_ = (A_1_τ_1_^2^ + A_2_τ_2_^2^)/(A_1_τ_1_ + A_2_τ_2_), where τ_1_, τ_2_ are the fluorescent lifetime and A_1_, A_2_ are pre-exponential factors.^33,34^ The τ_ave_ increases from 1.10 ns in CdS-0 to 1.88 ns in CdS-150. The longer τ_ave_ of CdS-150 indicates that the photocatalysts with a higher amount of coexposed (100) and (220) facets prolong the residence time of photogenerated charge carriers on CdS-150 surfaces, which increases the interaction between charge carriers and lignin. Both PL and TRPL results indicate that the phase junction CdS-150 with highest amount of coexposed hexagonal (100) and cubic (220) facets significantly prolongs the recombination time of photogenerated charge carriers and enhances the interaction between charge carriers and reactant, therefore improving the photocatalytic performance.

**Figure 4:**
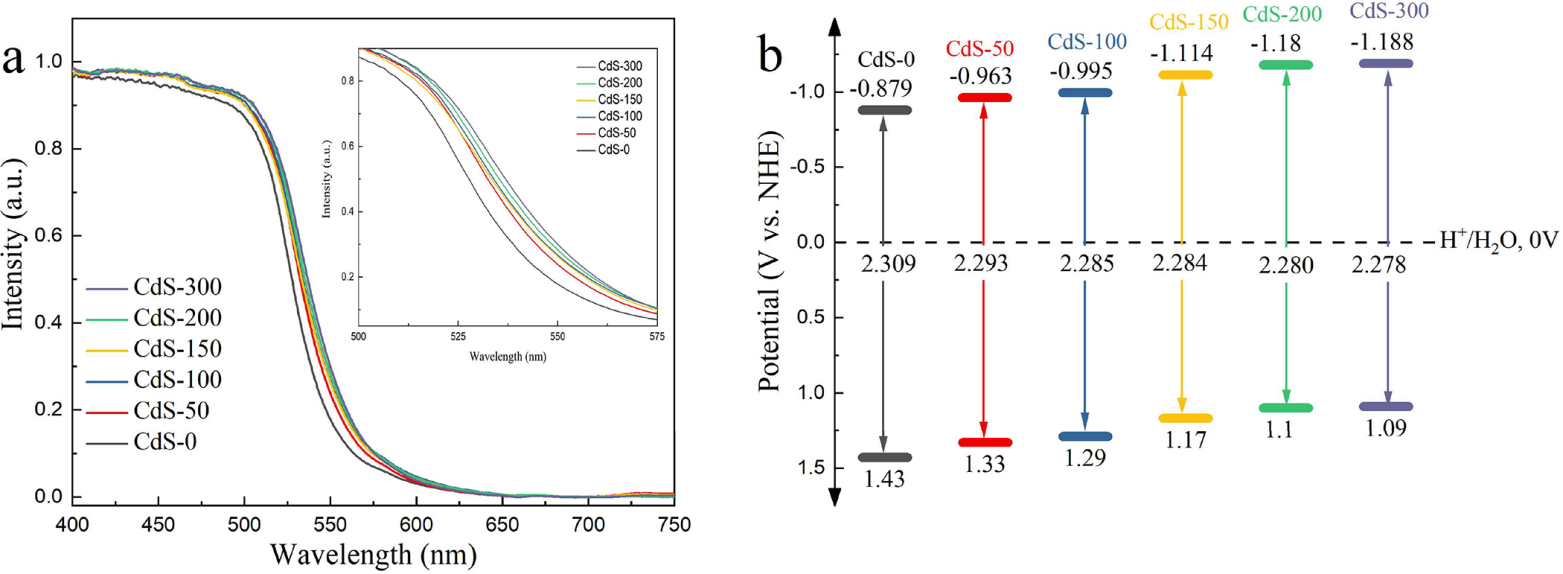
(a) UV–vis diffuse reflectance spectra and (b) band structure diagram of CdS-*n*. The inset graph in (a) is the magnified graph of the DRS results from 500 to 575 nm.

The visible light absorption capability, energy band positions, and photoredox capability of CdS photocatalysts are critical factors in photocatalytic conversion of lignin to aromatic monomers, and they could be optimized through adjusting the amount of coexposed hexagonal (100) and cubic (220) facets in phase junction CdS photocatalysts. DRS was conducted to evaluate the visible light absorption capability of phase junction CdS. In Figure 4a, DRS results show a slightly red shift from CdS-0 to CdS-300, indicating that solar-light-harvesting efficiency is gradually improved with an amount of coexposed hexagonal (100) and cubic (220) facets in phase junction CdS photocatalysts. Generally, the improved visible light absorption capability of photocatalysts is an important factor to facilitate the generation of charge carriers and then enhance the photocatalytic performance of lignin conversion.^31,35^ Moreover, the bandgap (E_g_) and valence band potential (E_VB_) were obtained based on DRS and XPS-VB results, which further determine the bandgap edge and photoredox capability of phase junction CdS photocatalysts (Figure S5 and S6). The band structure diagram for all photocatalysts (Figure 4b) shows the narrowed bandgap and negative shift of conduction band potential (E_CB_) and E_VB_ from CdS-0 to CdS-300. These results demonstrate that the phase junction CdS photocatalysts have a reductive capability and a weaker oxidative capability from CdS-0 to CdS-300. An appropriate photoredox capability of photocatalysts is an important factor to facilitate the cleavage performance of C_β_–O bonds to aromatic monomers.^7^ The excessive oxidative capability can lead to the overoxidation of lignin to PP-one byproduct, whereas the weak oxidative capability hinders the conversion rate of lignin to aromatic monomers. Based on the band structure of CdS photocatalysts, CdS-150 photocatalysts with the highest amount of coexposed hexagonal (100) and cubic (220) facets show an appropriate photoredox capability, therefore significantly improving the conversion rate of PP-ol and reducing the selectivity of PP-one byproduct.

### 3.3 Photocatalytic Performances of Cleaving C_β_–O Bonds in PP-ol

**Scheme 1:**
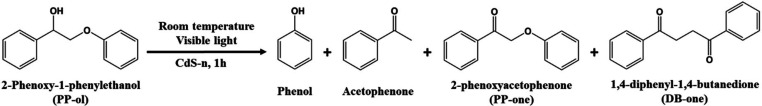
Photocatalytic Conversion of PP-ol to Phenol, Acetophenone, PP-one and DB-one

In this section, photocatalytic experiments were designed to investigate the impact of hexagonal CdS with exposed (100) facet, cubic CdS with exposed (220) facet, and phase junction CdS with coexposed hexagonal (100) and cubic (220) facets in the reaction rate of PP-ol and the selectivity of aromatic monomers and investigate the role of water as an external hydrogen donor to promote the cleavage of C_β_–O bonds in PP-ol. The dimeric lignin model (PP-ol) was first employed to evaluate the cleavage of C_β_–O bonds by photocatalysts, due to its interunit C–O bonds strongly resembles C_β_–O bonds in the real lignin biomass. Scheme 1 shows the photocatalytic cleavage of C_β_–O bonds in PP-ol and the obtained products, where acetophenone and phenol are the target monomeric aromatic products and dimeric aromatic compounds of PP-one and DB-one are not desirable products. PP-one as a byproduct is dehydrogenated from PP-ol, and DB-one is a byproduct from the C–C coupling side reaction.^7^ The blank experiments were also performed, and the results confirm that PP-ol cannot be converted without either CdS photocatalysts or visible light irradiation (Entries 1–2 in Table 1).

**Table 1:** Controlled Reaction Conditions for Fragmentation of C_β_–O Bonds^a^

				Conversion	Selectivity		
Entry	Catalyst	Atmosphere	Solvent	PP-ol	Phenol	Acetophenone	PP-one
1	No catalyst	Ar	CH_3_CN/H_2_O	-	-	-	-
2^b^	CdS-150	Ar	CH_3_CN/H_2_O	-	-	-	-
3	CdS-150	Air	CH_3_CN/H_2_O	43%	5.2%	5.3%	92.8%
4	CdS-150	O_2_	CH_3_CN/H_2_O	7%	-	-	100%
5	CdS-150	Ar	CH_3_CN/H_2_O	100%	94.3%	93.4%	3.6%
6	CdS-150	He	CH_3_CN/H_2_O	100%	93.3%	92.7%	5.8%
7	CdS-150	N_2_	CH_3_CN/H_2_O	100%	92.7%	89.8%	4.4%
8	CdS-150	Ar	CH_3_CN	21.8%	48%	42.4%	47.6%
9	CdS-150	Ar	Methanol	26.7%	76.3%	76.6%	17.2%
10	CdS-150	Ar	Ethanol	49.9%	60.8%	55.8%	31.5%
11	CdS-150	Ar	Isopropanol	42.8%	77.6%	71.9%	26.4%
12	CdS-150	Ar	0.5 mL of TEA and 4.5 mL of CH_3_CN	-	-	-	-
13^c^	CdS-150	Ar	CH_3_CN/H_2_O	-	-	-	-

a Typical reaction condition: lignin model compound PP-ol is 10 mg, photocatalyst is 10 mg, solvent (CH_3_CN/H_2_O (v/v = 3/7)) is 5 mL, Ar is at 1 atm, visible light power is 0.35 W cm^–2^, 1h.

b In the dark condition.

c Acetophenone as the reactant.

The presence of O_2_ in the reaction system could lead to the oxidation of PP-ol to PP-one, which is negative to the overall performance. As presented in Entries 3–4 in Table 1, the conversion rates of PP-ol and the selectivity of PP-one are 43% and 92.8%, respectively in the air atmosphere, whereas the conversion rate decreases to 7% and the product is only PP-one under pure O_2_ condition. There are two possible reasons to explain these results, one is that the formation of reactive oxygen species (e.g., ^•^O_2_^–^) can drive the oxidation of PP-ol to PP-one,^23^ and the other reason is that the presence of O_2_ can oxidize the hydrogen on the surface of photocatalysts and thus repress the hydrogenolysis of C_β_–O bonds.^7,23,36^ The selectivity of aromatic monomers and the conversion rate of PP-ol are similar in the inert atmospheres of Ar, He or N_2_ (Entries 5–7 in Table 1). These results indicate that the C_β_–O bond cleavage reaction should be performed in the absence of oxygen to avoid the side reactions.

#### 3.3.1 Improve PP-ol Conversion Rate through Enhanced Activation of C_α_–H Bonds and Fast Hydrogen Transfer Efficiency by Coexposed (100) and (220) Facets in Phase Junction CdS Nanoparticles

**Figure 5:**
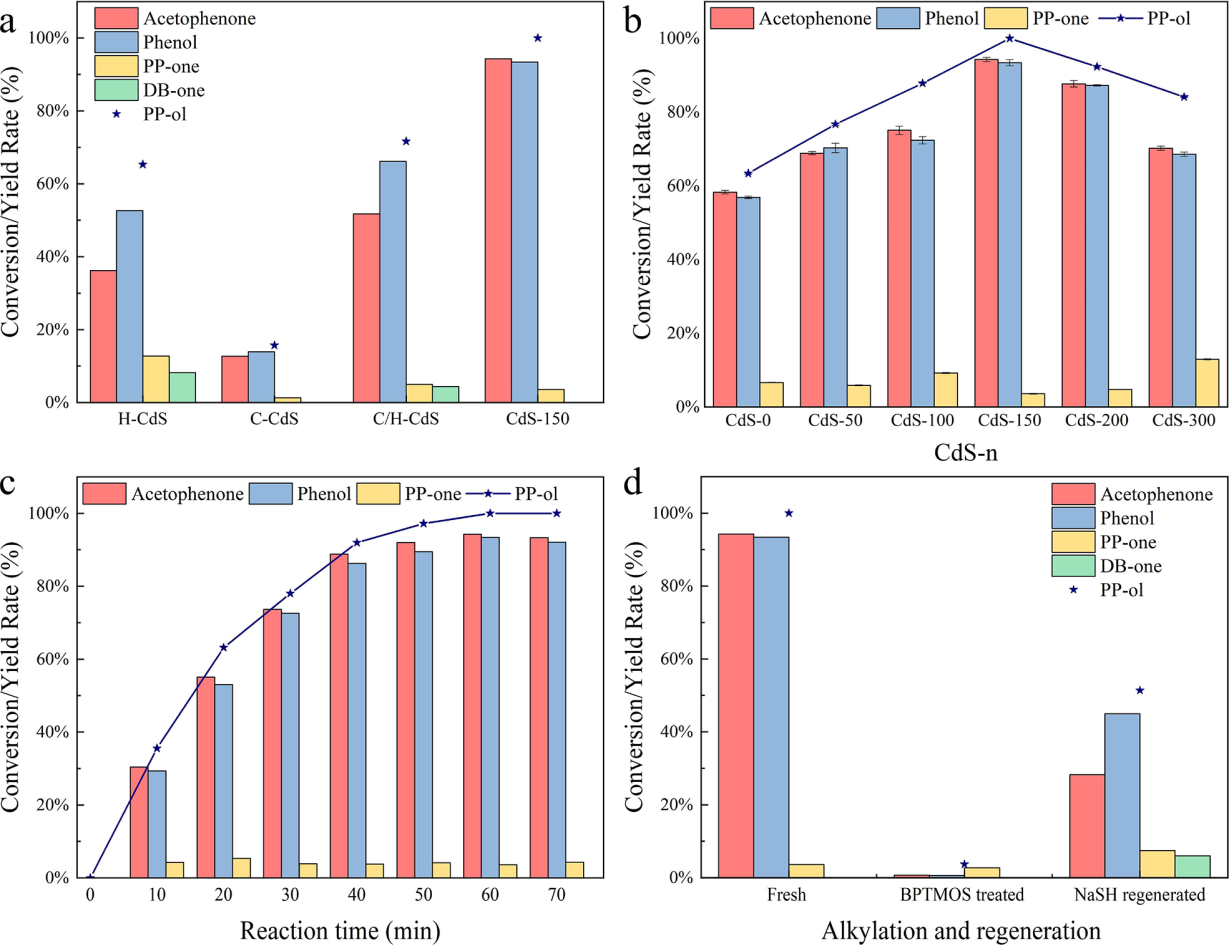
Conversion rate of PP-ol and the yields of products with (a) hexagonal CdS (H-CdS), cubic CdS (C-CdS), physically mixed C-CdS and H-CdS (C/H-CdS), and phase junction CdS (CdS-150), (b) CdS-*n*, (c) effect of reaction time using CdS-150, and (d) BPTMOS treated and NaSH regenerated CdS-150. Reaction condition: lignin model compound PP-ol is 10 mg, photocatalyst is 10 mg, solvent (CH_3_CN/H_2_O (v/v = 3/7)) is 5 mL, Ar is at 1 atm, visible light power is 0.35 W cm^–2^, 1 h.

The coexposed cubic (220) facet and hexagonal (100) facets can significantly improve the activation of C_α_–H bonds in the cleavage of C_β_–O bonds and improve the hydrogen transfer efficiency in reduction of DB-one production as C–C coupling byproduct. To understand the role of two facets in the PP-ol conversion, the cubic CdS (C-CdS) with exposed (220) facet, the phase junction CdS (CdS-150) with coexposed (100) and (220) facets, and the hexagonal CdS (H-CdS) with an exposed (100) facet were prepared to investigate the respective impacts on photocatalytic performance of lignin conversion. XRD results in Figure S7 confirm the successful synthesis of C-CdS, CdS-150, and H-CdS. These three photocatalysts were employed to investigate the photocatalytic performance of lignin conversion to aromatic monomers. As shown in Figure 5a, the H-CdS photocatalyst converts 65.3% of PP-ol to 52.6% of phenol and 36.2% of acetophenone as the desirable aromatic monomers, but 8.2% of DB-one is generated as the C–C coupling byproduct. In contrast, the C-CdS photocatalyst exhibits a relatively low PP-ol conversion of 15.7% and no production of DB-one. In the cleavage of C_β_–O bonds process, the generation of DB-one is mainly due to the low hydrogen transfer efficiency on the surface of photocatalysts, while the slow reaction rate is attributed to the low C_α_–H bonds activation by photocatalysts. Based on these results, we found that the activation of C_α_–H bonds in PP-ol can be mainly improved by the hexagonal (100) facet to cleave the C_β_–O bonds in PP-ol and the hydrogen transfer efficiency can be mainly improved by the cubic (220) facet to facilitate the generation of acetophenone and phenol. To further evaluate the impact of two crystal facets combined in a single crystal particle on the photocatalytic performance in lignin conversion, physically mixed C-CdS and H-CdS (C/H-CdS) have also been used to cleave the C_β_–O bonds. The results indicate that physically mixed C/H-CdS photocatalyst converts only 71.7% of PP-ol into 51.7% of acetophenone and 66.2% of phenol as desirable products, and produces 5% of PP-one and 4.4% of DB-one as byproducts. The physically mixed C/H-CdS cannot form a tight interface between the hexagonal (100) and cubic (220) facets. Therefore, it cannot effectively transfer photogenerated electrons from the hexagonal (100) facet to the cubic (220) facet. The prepared CdS-150, featuring a close interface between (100) and (220) facets, can achieve a complete conversion of PP-ol to around 94% of aromatic monomers after 1 h of visible light irradiation. Based on these results, CdS-150 photocatalysts can form a close interface between (100) and (220) facets, which can significantly improve the transfer efficiency of photogenerated electrons from hexagonal (100) facet to cubic (220) facet (Figure 3), therefore significantly improving the C_α_–H bonds’ activation to enhance the cleavage of C_β_–O bonds in lignin on (100) facet and dramatically improving the hydrogen transfer efficiency to decrease the generation of DB-one byproduct on (220) facet.

The conversion rate to aromatic monomers can be improved by increasing the amount of coexposed (100) and (220) facets in phase junction CdS photocatalysts, as improving photoexcited charge carriers’ migration efficiency, supplying appropriate photoredox capability, and increasing the number of active sites with the available coexposed (100) and (220) facets. The number of coexposed (100) and (220) facets in phase junction CdS photocatalysts can be optimized by the controlled ratio of Cd^2+^/S^2–^ through different amount of trisodium citrate in the hydrothermal process. XRD results demonstrate that the amount of coexposed (100) and (220) facets is increasing from CdS-0 to CdS-150 and CdS-150 has the highest amount of coexposed (100) and (220) facets (Figures 1c-d). Experimental results further confirm the above discussions. As shown in Figure 5b, the conversion rate of PP-ol is significantly improved from 63% to 100% from CdS-0 to CdS-150 photocatalysts after 1 h of visible light irradiation. However, a decreased conversion rate of PP-ol is observed in the CdS-200 and CdS-300 photocatalysts. Phase junction CdS-150 photocatalyst shows the best photocatalytic performance, in which PP-ol is completely converted to around 94% of acetophenone and phenol with 1 h of visible light irradiation (Figure 5c). This result shows the fastest reaction rate and the highest selectivity to desirable products compared to those of earlier works (Table S1). Both the calculated crystal sizes of the hexagonal and cubic phases and the calculated texture coefficient of cubic (220) and hexagonal (100) facets (Figures 1c-d) show that CdS-150 photocatalysts have the highest amount of coexposed (100) and (220) facets. The highest amount of coexposed (100) and (220) facets can improve the photoexcited charge carriers’ transfer efficiency (PEC, PL, and TRPL results in Figure S3 and S4) and provide appropriate photoredox capability (DRS results in Figure 3), therefore improving the conversion rate of PP-ol to aromatic monomers. Furthermore, BET results confirm that CdS-150 has the highest specific surface area (41.3 m^2^/g) and the highest pore volume (0.3 cm^3^/g) compared with other samples (Figure S8), which provide more active sites to improve the photocatalytic performance of CdS-150 than others.

The sulfur moieties on the surfaces of coexposed (100) and (220) facets in phase junction CdS can act as the active sites for improving the C_α_–H bonds activation in PP-ol, thereby enhancing the reaction rate of cleaving C_β_–O bonds to aromatic monomers. To prove the role of sulfur moieties as the active sites in photocatalytic performance, the alkylation and regeneration of CdS-150 were conducted to inhibit and restore the sulfur moieties on the surfaces of phase junction CdS, respectively.^14,37,38^ The fresh CdS-150 is alkylated with BPTMOS as the bromide derivatives in cyclohexane solution that can efficiently interact and inhibit the sulfur moieties on the surface of photocatalyst. As shown in Figure 5d, the BPTMOS treated CdS-150 exhibits a significant decrease in the conversion rate of PP-ol from 100% for the fresh photocatalyst to 3.7%, as well as a decreased selectivity to aromatic monomers from around 94% to around 15%. This result demonstrates the crucial role of sulfur moieties on the surfaces of phase junction CdS in enhancing the C_α_–H bonds activation of PP-ol to improve the reaction rate. To reactivate the photocatalysts, the BPTMOS treated CdS-150 is further regenerated by soaking in NaSH aqueous solution to remove BPTMOS groups. After the regeneration, the conversion rate of PP-ol is increased to 51.4%, and the selectivity of aromatic monomers is also largely recovered. However, DB-one is produced via the regenerated CdS-150, indicating that the sulfur moieties on the coexposed (100) and (220) facets is not completely restored through the regeneration process.^25^ The incompletely restored sulfur moieties on the CdS-150 trigger the poor hydrogen transfer efficiency to generate DB-one as the C–C coupling byproduct. The exposed sulfur moieties on the surface of (100) and (220) facets is particularly important in trapping holes for the C_α_–H bonds’ activation and hydrogen transfer efficiency to improve the reaction rate of PP-ol conversion and selectivity to aromatic monomers.^14,37,38^

#### 3.3.2 Enhance the Selectivity of Aromatic Monomers through Sufficient External Hydrogen Source

In the cleavage reaction of C_β_–O bonds, the external hydrogen supply determines the reaction rate and selectivity to acetophenone and phenol. PP-ol itself can supply the hydrogen for the generation of aromatic monomers through the self-hydrogen transfer process.^7^ However, in the CH_3_CN system (Entry 8 in Table 1), the conversion rate of PP-ol is only 21.8% and the selectivity of acetophenone and phenol are only 42.4% and 48%, respectively, after 1 h of visible light irradiation. The high yields of PP-one byproduct is due to insufficient supply of hydrogen and overoxidation of PP-ol to PP-one by photogenerated holes. A proper external hydrogen donor can improve the conversion rate and selectivity to desirable products. Methanol, isopropanol and ethanol are external hydrogen donors to improve the photocatalytic performance in the cleavage of C_β_–O bonds as shown in Entries 9–11 in Table 1. The addition of methanol, 2-propanol, or ethanol can increase the conversion rate of PP-ol to 26.7%, 42.8%, and 49.9% and decrease the selectivity of PP-one to 17.2%, 26.4%, and 31.5%, respectively. However, the conversion rate to aromatic monomers is still relatively low. Although TEA can supply hydrogen, the C_β_–O bonds in PP-ol are barely cleaved, which may be owing to its strong trapping capability for photogenerated holes (Entry 12 in Table 1).^39^

**Figure 6:**
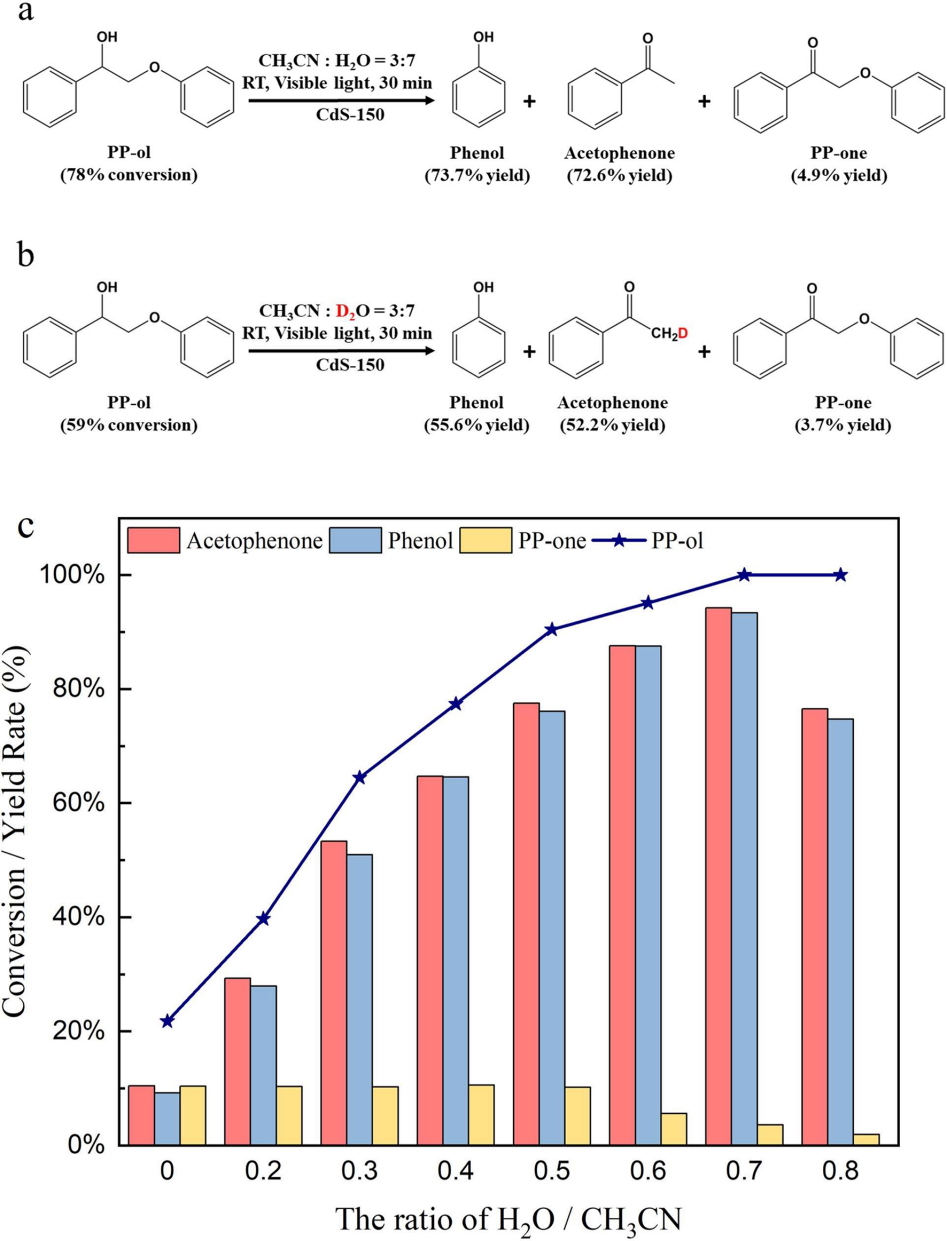
Photocatalytic conversion of PP-ol in (a) H_2_O–CH_3_CN and (b) D_2_O–CH_3_CN solvent conditions after 30 min of visible light irradiation. (c) Conversion rate of PP-ol and the yields of products with effect of water amount in the system using CdS-150. Reaction condition: lignin model compound PP-ol is 10 mg, CdS-150 is 10 mg, solvent (CH_3_CN/H_2_O(D_2_O) (v/v = 3/7)) is 5 mL, Ar is at 1 atm, visible light power is 0.35 W cm^–2^.

Water was found as an ideal external hydrogen source to significantly improve the conversion rate of PP-ol and to enhance the selectivity of aromatic monomers. In this study, water was used as an excellent hydrogen donor to significantly promote the reaction rate and selectivity to desirable products through the photocatalytic water dissociation process. The ratio of water in the reaction solvent can significantly affect the reaction performances, including the reaction rate and the selectivity to each product. As shown in Figure 6c, increasing the ratio of H_2_O in the solution system from 0 to 0.7 could improve the conversion rate of PP-ol from 21.8% to 100% and increase the yields of desirable products from 48% to 94%. These results indicate that H_2_O facilitates the conversion rate of PP-ol and improves the selectivity of the desirable aromatic monomers. Moreover, apart from the role of a hydrogen donor, the low solubility of chemicals in water can also increase the chance for PP-ol to linger on the surface of photocatalysts, thereby improving the conversion rate of PP-ol.^16^ When the ratio of water in solvent reaches 80%, PP-ol can be completely converted, but the products in the system fail to reach a stoichiometric balance. Some suspended matter was observed in the unbalanced solution after the photocatalytic reaction because of the low solubility of chemicals in the excess amount of water in the photocatalytic system (Figure S9). It has been reported that the wettability of the photocatalysts is an important material property for photocatalytic water splitting. The hydrophilic surface can increase the contact between H_2_O and the surface of CdS, therefore promoting the surface reaction between the water and photocatalyst.^40^ As shown in Figure S10, the contact angle between the water and CdS-150 is 39.91°, while the contact angle of water to CdS-0 is 46.22°. The better hydrophilic surface of CdS-150 increases its contact with water and promotes the photocatalytic performance.^41,42^

The addition of water to the system can supply hydrogen in the formation of acetophenone. An isotopic experiment of H_2_O/D_2_O was employed to further investigate the role of H_2_O in the cleavage of C_β_–O bonds and confirmed that water could provide sufficient hydrogen to promote the photocatalytic C_β_–O bond hydrogenolysis through the water dissociation process. To exclude the isotope exchange involved in this reaction, acetophenone and phenol are first tested with D_2_O under the typical reaction condition, and the results confirm that H/D exchange is not observed (Figure S11). As shown in Figures 6a-b, the conversion rate of PP-ol is 78% in the H_2_O system after 30 min reaction, while 59% of PP-ol is converted in the D_2_O system. The MS spectra of the products in Figure S12 show that the MS peak of acetophenone in the D_2_O system shifts from 120 to 121 *m*/*z*, indicating that one hydrogen atom in acetophenone molecule is replaced by deuterium. In addition, the MS peak at 43 *m*/*z* shifts to 44 *m*/*z*, which corresponds to the effect of O=C–CH_3_(CH_2_D). The MS results confirm that the location of deuterium is in its methyl group.^36^ The formation of deuterated acetophenone indicates the hydrogen transfer from water to the acetophenone during the C_β_–O bond hydrogenolysis process. Besides, the MS signal of phenol in the two systems of H_2_O and D_2_O are the same, indicating that the hydrogen in the hydroxyl group of phenol is obtained from the reactant via the self-hydrogen transfer mechanism which was also reported in the literature.^7^

#### 3.3.3 Photostability and Feasibility of Coexposed (100) and (220) Facets in Phase Junction CdS for Lignin Conversion to High-Value Aromatic Monomers

**Figure 7:**
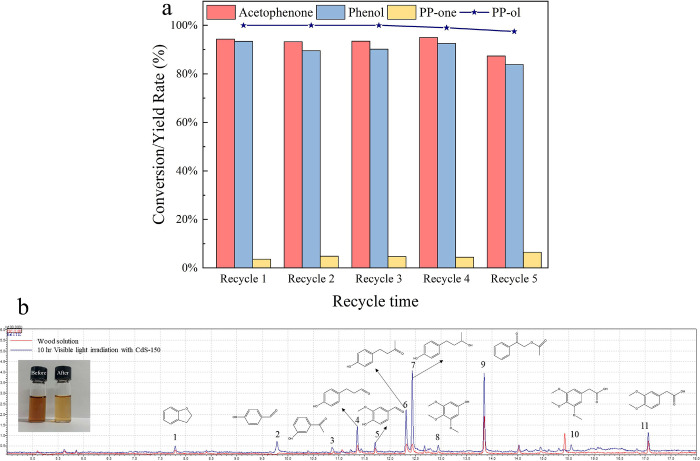
(a) Conversion rate of PP-ol and the yields of products with a reusability test using CdS-150 after 1 h of visible light irradiation. (b) GC-MS spectra for photocatalytic converted products of wood extraction lignin solution. The solution color before and after the photocatalytic reaction (inset graph). Reaction conditions: wood extraction powders are 80 mg, CdS-150 is 20 mg, H_2_O and CH_3_CN mixed solution (CH_3_CN/H_2_O (v/v = 3/7)) is 7 mL, Ar is at 1 atm, visible light power is 0.35 W cm^–2^, 10 h.

Excellent photostability and feasibility are important factors in the large-scale application of photocatalytic lignin fragmentation to high-value aromatic monomers. To evaluate the photostability of the CdS-150 photocatalyst, we conducted five consecutive recycling tests. After each cycle, the used photocatalyst was washed three times with CH_3_CN to remove any residual adsorbed chemicals and then dried in a vacuum oven at 60 °C for 2 h. These results indicate that the photocatalytic performance of CdS-150 remains relatively stable during the 5 cycles as shown in Figure 7a. The XRD pattern of the recycled sample exhibits similar diffraction peaks between fresh and recycled CdS-150 (Figure S13). Overall, these results indicate that CdS-150 has a high photostability during the photocatalytic reaction process.

**Scheme 2:**
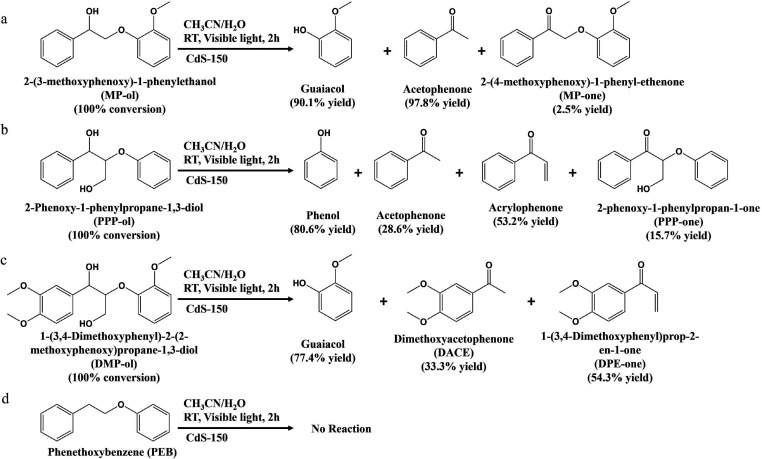
Conversion of Different Lignin Models to Desirable Aromatic Monomers after 2 h of Visible Light Irradiation. (a) MP-ol; (b) PPP-ol; (c) DMP-ol; (d) PEB Reaction conditions: lignin model compounds is 10 mg, CdS-150 is 10 mg, solvent (CH_3_CN/H_2_O (v/v = 3/7)) is 5 mL, Ar is at 1 atm, visible light power is 0.35 W cm^–2^, 2 h.

Various lignin models were conducted to investigate the feasibility of the CdS-150 photocatalyst in lignin conversion to aromatic monomers. The methoxy group is a major constituent of real lignin. Thus, MP-ol, with a methoxy-substituted structure, was used as the lignin model compound to investigate the feasibility of the CdS-150 for lignin conversion. The result (Scheme 2a and Figure S14) indicates that MP-ol is completely converted via CdS-150 with the support of H_2_O. The converted products contain 97.8% of acetophenone and 90.1% of guaiacol as the desirable products and only 2.5% of MP-one is generated as the byproduct after 2 h of visible light irradiation. The native β-O-4 motif in real lignin contains not only a benzylic hydroxyl group at the α position (secondary benzylic alcohol, such as PP-ol) but also a hydroxymethyl group at the β position (primary aliphatic alcohols).^43−45^ Herein, PPP-ol, which contains a benzylic hydroxyl group and a hydroxymethyl group, was used to investigate the feasibility of the CdS-150 photocatalyst in lignin conversion to aromatic monomers. As shown in Scheme 2b and Figure S14, PPP-ol with both key functional groups can be completely converted to 80.6% of phenol, 28.6% of acetophenone and 53.2% of acrylophenone as desirable aromatic monomers after 2 h of visible light irradiation. A more complex lignin model compound DMP-ol, which contains a benzylic hydroxyl group, a hydroxymethyl group, and three methoxy groups, was also used to further demonstrate the feasibility of our developed CdS-150 photocatalysts in the cleavage of C_β_–O bonds in lignin to aromatic monomers. As shown in Scheme 2c and Figure S14, DMP-ol can be completely converted to 77.4% of guaiacol, 33.3% DACE and 54.3% DPE-one as desirable aromatic monomers after 2 h of visible light irradiation.

The cleavage of C_β_–O bonds in different lignin model compounds can help us fully understand the effect of the chemical structure role of substitutes, such as benzylic hydroxyl groups at the α position (C_α_–OH groups), hydroxymethyl group at the β position (C_β_-C_γ_–OH groups), and the methoxy groups, in the conversion of lignin to aromatic monomers. Based on the above-mentioned results, the effect of three substitutes in conversion of lignin models is discussed below. First, in the cleavage of C_β_–O bonds in different lignin model compounds, the C_α_–OH groups play an important role in the generation of aromatic monomers, as the activation of C_α_–H bonds can significantly reduce the bond dissociation energy (BDE) of C_β_–O bonds from 55 kcal/mol in PP-ol to 7.8 kcal/mol,^4^ therefore improving the conversion rate of lignin to aromatic monomers. To investigate the importance of C_α_–OH groups in lignin, the PEB lignin model without C_α_–OH groups was conducted, but no products were generated after 2 h of visible light irradiation (Scheme 2d), indicating that the activation of C_α_–H bonds only happens in the presence of C_α_–OH groups. Second, the reaction time in complete conversion of lignin model compounds (Scheme 2a-c), including MP-ol, PPP-ol and DMP-ol, is longer than that of PP-ol under visible light irradiation. The longer reaction time in complete conversion of these lignin model compounds compared to that of PP-ol was also demonstrated in previous works, but they did not investigate the reason for the slow reaction rate.^4,7,13,15,17,18^ The slower reaction rate might be attributed to the presence of electron-donating groups (EDG), such as methoxy substituents and C_γ_–OH groups in lignin model compounds, which increase the electron density of lignin model compounds and decrease the adsorption capability of lignin model compounds on the photocatalysts’ surfaces,^46,47^ therefore prolonging the reaction time. This assumption can be proved by the photocatalytic reductive coupling of the aryl bromides reaction. For example, Zheng’s group demonstrated that the presence of EDG in aryl bromides can decrease the yields of targeted products after the same reaction time (20 h of 420 nm LED irradiation) compared to that of aryl bromides without EDG, as the EDG can increase the electron density of the bromobenzene moiety to suppress the single electron transfer from photocatalysts to substrates by decreasing the electron-accepting capacity of substrates.^46^ Third, in our reaction system, the presence of C_β_-C_γ_–OH groups in PPP-ol and DMP-ol lignin model compounds can affect the final acetophenone-like products. For example, the conversion of PPP-ol generates acetophenone and acrylophenone as acetophenone-like products, and the conversion of DMP-ol generates DACE and DPE-one as acetophenone-like products. However, in previous works, as shown in Scheme S1, the conversion of PPP-ol and DMP-ol would typically generate hydroxypropiophenone (HPP) and 1-(3,4-dimethoxyphenyl)-3-hydroxypropan-1-one (DPH-one) as acetophenone-like products, respectively, both containing C_γ_–OH groups.^4,13,16−18^ In our reaction system, the generation of acetophenone and DACE are the products after the cleavage of C_β_-C_γ_ bonds in HPP and DPH-one, respectively, as well as acrylophenone and DPE-one are generated after the dehydration of C_γ_–OH groups in HPP and DPH-one, respectively. Both cleavage of C_β_-C_γ_ bonds and dehydration of C_γ_–OH groups in HPP and DPH-one to generate acetophenone-like products in our reaction system might be attributed the C_γ_–OH groups activation. This assumption can be proved by the photocatalytic dehydration of ethanol to ethylene through C–OH groups activation.^48,49^ For example, Ma’s and Li’s groups have demonstrated that tungsten oxide (WO_3–*x*_) and tungsten oxide with carbon coating (WO_3–*x*_@C), abstracted hydrogen from C–OH groups in ethanol to form C_2_H_5_O^•^ radical intermediates, and then the C–O bonds were easily cleaved to generate ethylene.^48,49^ Hence, in the conversion of both lignin model compounds, the C_γ_–OH groups are activated to form C_γ_–O^•^ radical intermediates through photogenerated holes, therefore generating the acetophenone-like products with C_β_=C_γ_ groups, such as acrylophenone and DPE-one. Also, the formed C_γ_–O^•^ radical intermediates may trigger the cleavage of C_β_-C_γ_ bonds to generate acetophenone in the conversion of PPP-ol and DACE products in the conversion of DMP-ol.

To further evaluate the developed photocatalytic process and CdS-150 photocatalysts for real lignin conversion, we tested the fragmentation of lignin extracted from the wood sawdust was tested. The GC-MS was used to identify the products in the lignin solution both before and after 10 h of visible light irradiation. The results are presented in Figures 7b and S15 and demonstrated that the converted products consist of 11 distinct value-added aromatic monomers in real lignin solution and the intensity peaks corresponding to 4, 6, and 7 are approximately 3–5 times as their level before the fragmentation of lignin. The increased intensity of these peaks shows significant enhancement in the selectivity of aromatic monomers after 10 h of visible light irradiation. The change in the color of the lignin solution after 10 h of visible light irradiation further confirms the effective conversion of real lignin into aromatic monomers under the CdS-150 photocatalyst. Overall, the phase junction CdS is a photocatalyst with a high potential for lignin conversion into valuable aromatic monomers.

#### 3.3.4 Mechanism of C_β_–O Bonds Cleavage in PP-ol

Photogenerated charge carriers, including holes and electrons, can transfer from CdS photocatalysts to PP-ol to improve the C_α_–H bonds’ activation, therefore enhancing the cleavage of C_β_–O bonds in PP-ol to aromatic monomers. To investigate the charge carriers’ transfer, PL analysis of CdS-150 photocatalysts was conducted at varying PP-ol concentrations. As shown in Figure S16a, the emission intensity of the excited state of CdS is gradually decreased with increasing PP-ol concentrations. Generally, a lower emission intensity represents a higher photogenerated charge carriers’ separation efficiency. The Stern–Volmer quenching constant of PP-ol (*K*_SV(PP-ol)_) was further calculated through the Stern–Volmer eq (Supporting Information). Figure S16b shows the linear Stern–Volmer behavior and the calculated *K*_SV(PP-ol)_ at 0.03 mM^–1^ with the *R*^2^ at 0.9792. Both results of lower emission intensity with the increased concentration of PP-ol and the linear Stern–Volmer behavior with *K*_SV(PP-ol)_ of 0.03 mM^–1^ indicate that the recombination time of photogenerated charge carriers is prolonged in the presence of PP-ol, and the photogenerated holes and electrons transfer from CdS photocatalysts to PP-ol, therefore improving the C_α_–H bonds activation and enhancing the conversion rate of PP-ol to aromatic monomers.

To further understand the roles of photogenerated holes and electrons in the cleavage of C_β_–O bonds in PP-ol, the hole scavengers and electron scavengers were separately added into the lignin conversion system. The addition of the hole scavengers can completely suppress the conversion of PP-ol (Entry 1 in Table 2), indicating that the photogenerated holes are involved in the first step of the C_α_–H bonds activation.^14^ The subsequent controlled experiment with the addition of electron scavengers results in a slight decrease in the conversion rate of PP-ol and a significant decrease in the selectivity of acetophenone and phenol (Entry 2 in Table 2), indicating that the cleavage of C_β_–O bonds is highly dependent on the photoexcited electrons. These results confirm that the photogenerated charge carrier transfer between CdS and PP-ol can highly determine the cleavage of C_β_–O bonds in PP-ol. Specifically, photogenerated holes can facilitate the first step of the C_α_–H bonds activation in PP-ol, while the photoexcited electrons can enhance the cleavage of C_β_–O bonds to aromatic monomers.

**Table 2:** Controlled Experiments by CdS-150 with Different Scavengers^a^

		Conversion	Yield		
Entry	Condition	PP-ol	Acetophenone	Phenol	PP-one
1	Hole scavengers	-	-	-	-
2	Electron scavengers	96.6%	16.5%	16.5%	80%
3	Radical scavengers	10%	5.4%	8.6%	3.8%

a Reaction condition: PP-ol is 10 mg, photocatalyst is 10 mg, solvent (CH_3_CN/H_2_O (v/v = 3/7)) is 5 mL, Ar is at 1 atm, visible light power is 0.35 W cm^–2^, 1 h. Hole scavengers: 20 mg of Na_2_S and 10 mg of Na_2_SO_3_; electron scavengers: 30 mg of Na_2_S_2_O_8_; radical scavengers: 30 mg of DMPO.

The generation of C_α_ radical intermediates also plays an important role in determining the selectivity of aromatic monomers, as the C_α_ radical intermediates can significantly decrease the BDE of C_β_–O bonds from 55 kcal mol^–1^ in PP-ol to 7.8 kcal mol^–1^.^4^ As shown in Entry 3 in Table 2, the addition of radical scavengers leads to a significant decrease in the conversion rate of the reactant (10%) and the yields of aromatic monomer chemicals (5.4% of acetophenone and 8.6% of phenol). This result implies that the formation of radical intermediates in PP-ol is essential to generate desirable aromatic monomers.

**Scheme 3:**
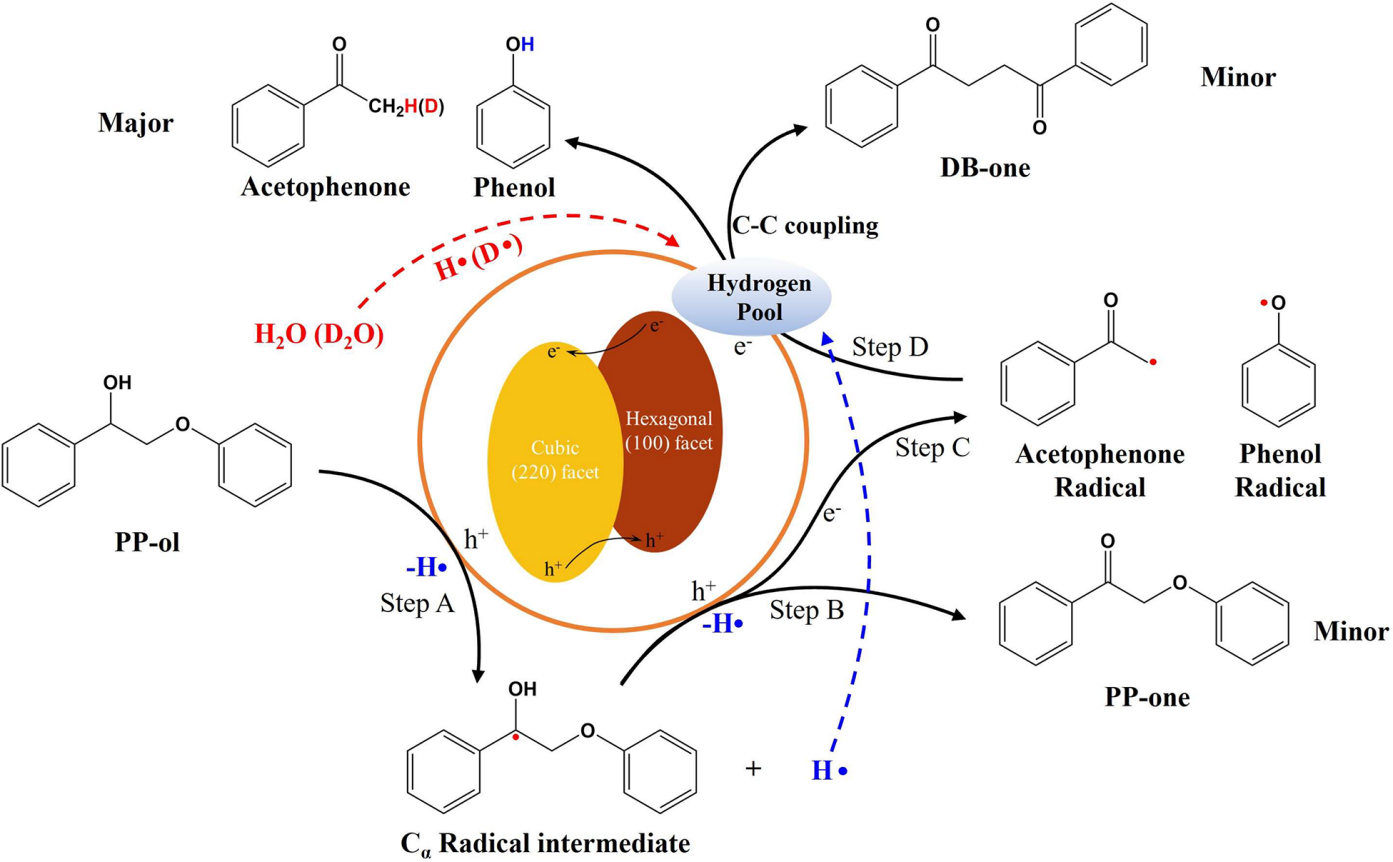
Proposed Mechanism of C_β_–O Bonds’ Fragmentation in Photocatalytic Conversion of PP-ol over Phase Junction CdS Photocatalyst

Phase junction CdS-150 with the highest amount of coexposed (220) and (100) facets photocatalysts can improve the migration efficiency of photogenerated electrons from hexagonal (100) facet to cubic (220) facet, therefore significantly improve the C_α_–H bonds activation in lignin on (100) facet and enhance the hydrogen transfer efficiency on (220) facet. Based on all above-mentioned results, the mechanism of C_β_–O bonds cleavage in lignin by phase junction CdS photocatalysts with coexposed (220) and (100) facets is proposed in Scheme 3. Under the visible light irradiation, photogenerated holes (h^+^) transfer from (220) facet to (100) facet and provide the photo-oxidation capability on (100) facet, while the photogenerated electrons (e^–^) transfer from (100) facet to (220) facet and provide the photoreduction capability on (220) facet. In detail, the photocatalytic C_β_–O bond cleavage consists of four steps. In step A, the h^+^ drives the oxidation of PP-ol to generate C_α_ radical intermediates and hydrogen on the (100) facet. The extracted hydrogen is adsorbed on the surfaces of phase junction CdS photocatalysts to form “hydrogen pool” for the subsequent steps.^50,51^ However, in Step B, the C_α_ radical intermediates can be further oxidized by h^+^ to generate PP-one as the byproduct.^7,14^ As shown in Figure 6, the addition of water can decrease the selectivity of PP-one byproduct, as water can consume excessive h^+^. In step C, the formed C_α_ radical intermediates in Step A can react with e^–^ and cleave the C_β_–O bonds to generate acetophenone radicals and phenol radicals on the (220) facet. In this step, the C_β_–O bonds in C_α_ radical intermediates are easily cleaved by the e^–^, because the BDE of C_β_–O bonds decreases from 55 kcal/mol in PP-ol to 7.8 kcal/mol in C_α_ radical intermediates.^4,15^ In Step D, the acetophenone radicals and phenol radials can obtain the hydrogen from the “hydrogen pool” on the surfaces of photocatalysts to generate target products. Acetophenone radicals can obtain the hydrogen from the dehydrogenation of water, whereas the proton in the hydroxyl group on phenol is obtained from the dehydrogenation reaction in Steps A and B. If the photocatalysts cannot supply fast hydrogen transfer efficiency for the formation of acetophenone, DB-one will be produced as byproduct through self C–C coupling of acetophenone radicals in step D. This C–C coupling reaction in the poor hydrogen transfer efficiency system can further reaffirm the importance of hydrogen transfer efficiency to the overall reaction performance of C_β_–O bonds cleavage. In summary, the phase junction CdS-150 photocatalysts with the highest amount of coexposed (220) and (100) facets significantly improve the conversion rate of lignin to aromatic monomers, as the coexposed (220) and (100) facets can form a close interface to enhance the charge carrier transfer from the (100) facet to (220) facet, therefore improving the C_α_–H bonds activation to enhance the cleavage of C_β_–O bonds in lignin on the (100) facet and facilitating the hydrogen transfer efficiency to decrease the generation of DB-one byproduct on the (220) facet.

### 3.4 Cleavage of C_β_–O Bonds in Ketone Byproduct through Assistance of TEA

PP-one is the byproduct in the above photocatalytic reaction. It is important to cleave the C_β_–O bonds in PP-one and improve the yields of acetophenone and phenol in the whole reaction. In the literature, PP-one was considered as an intermediate product, where PP-ol was first oxidized to PP-one by two photogenerated holes, the PP-one could be subsequently decomposed to aromatic monomers by the reduction driven from the photogenerated electrons.^6^ However, in this study, PP-one is not an intermediate product and can barely be converted in the CH_3_CN or H_2_O–CH_3_CN systems (Entries 1–2 in Table 3), as the cleavage of the C_β_–O bonds in PP-one requires a highly reductive photocatalyst and the participation of hydrogen donors.^51^ To further confirm this hypothesis, alcohols were first used as the hole sacrificial agent and hydrogen donor for the cleavage of β-O-4 ketones to see their effect on the reaction of PP-one.^6^ These results show that the conversion rate of PP-one is only 7.8% and 2.5% in ethanol and methanol systems, respectively (Entries 3–4 in Table 3). The relatively low conversion rate indicates that alcoholic solutions are the weaker hydrogen donor sources.^51−53^

**Table 3:** Effect of the Solvents on the Photocatalytic Claims of PP-one^a^

			Conversion	Yield	
Entry	Solvent condition	Reaction time (min)	PP-one	Acetophenone	Phenol
1	CH_3_CN	60	-	-	-
2	CH_3_CN/H_2_O	60	-	-	-
3	Methanol	60	2.5%	2.7%	2.1%
4	Ethanol	60	7.8%	7.7%	7.6%
5	0.5 mL of TEA into 4.5 mL of CH_3_CN/H_2_O	10	100%	95.1%	98.2%
6	0.5 mL of TEA into 5 mL of CH_3_CN	10	100%	50%	98.3%

a Reaction condition: PP-one is 10 mg, photocatalyst is 10 mg, mixed H_2_O and CH_3_CN (CH_3_CN/H_2_O (v/v = 3/7)) is 5 mL, Ar is at 1 atm, visible light power is 0.35 W cm^–2^.

Herein, a TEA meditated oxidation mechanism driven by photocatalysis was first proposed for the cleavage of the C_β_–O bonds in PP-one to understand the fast conversion rate and high selectivity to desirable aromatic monomeric products with assistance of TEA. In this process, the activation of the C_α_=O bonds in PP-one is an important step in the cleavage of the β-O-4 bonds. TEA can interact with weakly basic C_α_=O bonds to form a two-center/three-electron bonds with a lower energy demand for cleaving C_β_–O bonds.^54,55^ TEA can serve as both electron donor and proton donor in this reaction, and can effectively prevent the recombination of photogenerated holes and electrons and provide hydrogen for the formation of aromatic products.^39,55^ As demonstrated in Entry 5 in Table 3, the addition of TEA results in the rapid and complete conversion of PP-one to aromatic monomers after only 10 min of visible light illumination. From our best knowledge, the cleavage rate of C_β_–O bonds in ketone compound achieved in this study is the fastest among the reported works in the literature (Table S2).

Previous works have indicated that the TEA can be oxidized to amino radical cation TEA^+•^, and the TEA^+•^ can significantly improve the C_α_=O bonds activation in the ketone compound to facilitate the generation of desirable products, such as C–C coupling of acetophenone to pinacol.^54,55^ In these works, the photogenerated holes show high potential in transferring between CdS and TEA. To confirm the similar mechanism may exist in the cleavage of C_β_–O bonds in PP-one with assistance of TEA in our experiments, both PL analysis and K_SV_ calculation were conducted under the two conditions, (1) with PP-one but without TEA and (2) with PP-one and TEA, for comparing the quenching efficiency of CdS with two quenchers (TEA and PP-one). To better compare the *K*_SV(PP-one)_ and *K*_SV(TEA)_ in contributions of PP-one and TEA to quenching efficiency, the dimensionless ratios of TEA and PP-one are used to replace their actual concentration as the concentration of TEA (988 mM) is much higher than that of PP-one (9.42 mM) in our photocatalytic reaction system. The detailed information is presented in Figure S17 below. As shown in Figure S17a-b, the emission intensity of the excited state of CdS slightly decreases with the increasing PP-one weight, and the calculated *K*_SV(PP-one)_ was 0.0859 with *R*^2^ at 0.9186. The slightly lower *K*_SV(PP-one)_ represents that the photogenerated holes show the relatively low transfer efficiency between CdS and PP-one. In contrast, as shown in Figure S17c,d, the emission intensity of the excited state of CdS significantly decreases with the increasing volume of TEA and the calculated *K*_SV(TEA)_ is 0.374 with *R*^2^ at 0.989. Both Stern–Volmer plots of TEA and PP-one are linearity, representing the dynamic quenching between CdS and TEA as well as CdS and PP-one. The higher *K*_SV(TEA)_ compared to the *K*_SV(PP-one)_ represents that the photogenerated holes mainly transfer between CdS and TEA to generate TEA^+•^ and then the formed TEA^+•^ reacts with PP-one, therefore achieving the C_α_=O bonds activation in PP-one and converting PP-one to desirable aromatic monomers.

Interestingly, we noticed that water also acts as the hydrogen donor to enhance the formation of desirable products in this system, not only just TEA in the reaction system. As shown in Entry 6 in Table 3, the PP-one is completely converted in the anhydrous system, but the yield of acetophenone is only 50%, indicating that TEA cannot supply sufficient hydrogen for the formation of acetophenone. The H_2_O/D_2_O isotope labeling experiments conducted in CH_3_CN-D_2_O indicate that the hydrogen in the methyl group of acetophenone is replaced by deuterium from D_2_O, and the hydrogen in the hydroxyl group of phenol is still hydrogen (Figure S18). These results confirm that both water and TEA are the hydrogen sources to supply hydrogen for the generation of aromatic monomer products.

**Scheme 4:**
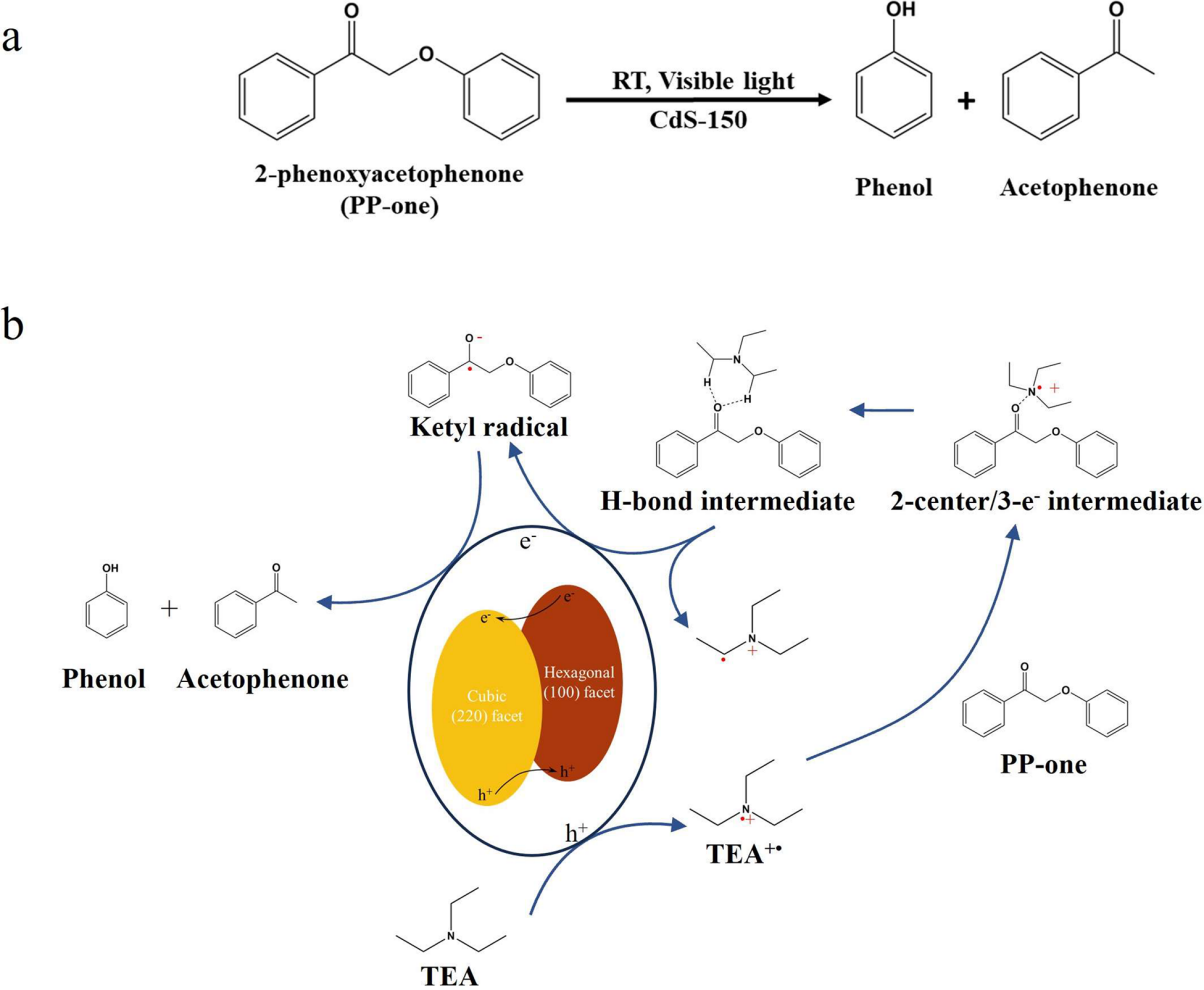
(a) Photocatalytic Conversion of PP-one to Phenol and Acetophenone. Reaction Condition: PP-one is 10 mg, Photocatalyst is 10 mg, Solvent (CH_3_CN/H_2_O (v/v = 3/7)) is 4.5 mL, TEA is 0.5 mL, Ar is at 1 atm, Visible Light Power is 0.35 W cm^–2^. (b) Postulated Reaction Pathways from PP-one to Acetophenone and Phenol

Based on the above discussions, a plausible mechanism regarding the cleavage of the C_β_–O bonds in PP-one is proposed with TEA as the mediator from this study. As shown in Scheme 4b, TEA is first oxidized by photoexcited holes to generate TEA^+•^ and proton (H^+^), and thereby facilitate the C_β_–O bonds hydrogenolysis reaction. The formation of TEA^+•^ can interact with weakly basic C_α_=O bond to form a two-center/three-electron intermediate.^54−56^ This intermediate is further isomerized to hydrogen bond intermediate and eventually formed the ketyl radical due to the lower energy barrier.^55^ Once the ketyl radical is formed, the C_β_–O bonds can be easily decomposed into phenol and acetophenone with the assistance of photogenerated electrons.^4,15^ Furthermore, H_2_O and TEA can act as the hydrogen sources in the formation of monomeric aromatic products.

## 4 Conclusion

The conversion rate of PP-ol to aromatic monomers can be significantly improved via the activation of C_α_–H bonds in PP-ol, enhanced hydrogen transfer efficiency, and sufficient external hydrogen supply from water dissociation. The coexposed hexagonal (100) and cubic (220) facets in phase junction CdS can significantly improve the photogenerated charge carriers’ migration efficiency and hydrogen transfer efficiency to improve the conversion rate of PP-ol and reduce the yields of DB-one as the C–C coupling byproduct. The hexagonal (100) and cubic (220) facets can expose sufficient sulfur moieties as the active sites. The alkylation and regeneration of sulfur moieties demonstrate that the sulfur moieties can facilitate the activation of C_α_–H bonds in PP-ol to form C_α_ radical intermediates that can enhance the conversion rate of PP-ol to aromatic monomers. Furthermore, water can act as an external hydrogen supplier to promote the formation of desirable aromatic monomers through water dissociation and reduce the generation of PP-one byproduct through consumption of the residue of photogenerated holes. The H_2_O/D_2_O isotopic labeled experiment confirms that the hydrogen for the formation of acetophenone is from water splitting. The PP-ol can be completely converted to ∼94% of acetophenone and phenol as desirable aromatic monomers via 1 h of visible light irradiation, which shows the best photocatalytic performance in comparison with the results reported in literature.

PP-one is the primary byproduct in PP-ol conversion and can be barely converted to the desirable aromatic monomers in the H_2_O–CH_3_CN system. We found that PP-one can be completely converted when TEA is added to the photocatalytic system. With the addition of TEA, the reaction time is significantly reduced from several hours reported in the literature to only 10 min of visible light irradiation. In this system, TEA not only acts as the hydrogen source and electron donor but also serves as the mediator to overcome the high reaction barrier of activation of C_α_=O bond in PP-one. In this reaction, TEA is first oxidized by photoexcited holes to generate TEA^+•^ and hydrogen for the C_β_–O bonds hydrogenolysis reaction. The TEA^+•^ interacts with weakly basic C_α_=O bonds in PP-one to reduce the energy barrier of the activation of C_α_=O bonds and facilitate the formation of a ketyl radical. The C_β_–O bonds in ketyl radical can be subsequently cleaved to acetophenone and phenol with assistance of photogenerated electrons. Overall, this study demonstrates that phase junction CdS is a promising photocatalyst candidate for the conversion of lignin to aromatic monomers, and the cleavage of C_β_–O bonds is facilitated by promoting key step reactions, including the activation of C_α_–H/C_α_=O bonds and the improvement in hydrogen transfer and supply.

## Supporting Information Available

SEM images; PEC properties, E_g_ and E_VB_, and BET results for CdS-*n* photocatalysts; PL and TRPL spectra, and contact angle of CdS-0 and CdS-150 photocatalysts; Full XPS spectrum of CdS-150; XRD patterns of C-CdS, H-CdS, and CdS-150; Photocatalytic performance in lignin conversion from previous works; The image of the reaction solution after 1h photocatalytic reaction; MS spectra of reactant and products after lignin conversion; Photoluminescence emission spectra of CdS with or without reactant (PDF)
