# Supplementary material for: Improved Lignin Conversion to High-Value Aromatic Monomers through Phase Junction CdS with Coexposed Hexagonal (100) and Cubic (220) Facets
Source: ACS Appl Mater Interfaces. 2024 Jun 4;16(23):29991–30009. doi: 10.1021/acsami.4c02315 (PMC11181269; doi:10.1021/acsami.4c02315)
Supplement: Supplementary file 1 — am4c02315_si_001.pdf [file am4c02315_si_001.pdf]

Supporting Information.

Improved Lignin Conversion to High-Value Aromatic Monomers through Phase Junction CdS with Co-Exposed Hexagonal (100) and Cubic (220) Facets

*Zongyang Yue [a, §], Shibo Shao [a, b, §], Jialin Yu [a], Guanchu Lu [a], Wenjing Wei [a], Yi Huang [a], Kai Zhang [c], Ke Wang [d, e, *], Xianfeng Fan [a, *]*

[a] Institute for Materials and Processes, School of Engineering, The University of Edinburgh, Edinburgh EH9 3BF, UK

[b] Petrochemical Research Institute, PetroChina Company Limited, Beijing 102206, China

[c] Beijing Key Laboratory of Emission Surveillance and Control for Thermal Power Generation, North China Electric Power University, Beijing 102206, China

[d] Beijing Key Laboratory of Ionic Liquids Clean Process, CAS Key Laboratory of Green Process and Engineering, State Key Laboratory of Mesoscience and Engineering, Innovation Academy

for Green Manufacture, Institute of Process Engineering, Chinese Academy of Sciences, Beijing 100190, China

[e] Longzihu New Energy Laboratory, Zhengzhou Institute of Emerging Industrial Technology, Henan University, Zhengzhou 450000, China

[*] Xianfeng Fan: Tel.: +441316505678; Email: x.fan@ed.ac.uk;

[*] Ke Wang: Tel.: +8613681127360; Email: kewang@ipe.ac.cn

[§] Z.Y. and S.S. contributed equally to this work.

Preparation of Hexagonal CdS

The hexagonal CdS was prepared by a one-pot hydrothermal method. Typically, 308 mg $Cd(NO_3)_2·4H_2O$ and 150 mg of trisodium citrate were dissolved into 15 ml of water and EG mixed solution ($v_{water}/v_{EG} = 1/5$). After being ultrasonically dispersed for 10 min and vigorously stirred for 30 min, 375 mg thioacetamide was added to the solution. After being stirred for another 30 min, the mixture was transferred into a 25 mL stainless Teflon-lined autoclave reactor. The autoclave reactor was subsequently heated to 160 ˚C with a 3 ˚C min^{-1} of heating rate in an oven and kept the temperature for 24h. After naturally cool-down, the sample was collected by centrifugation (9000 rpm), rinsed several times with ethanol and water respectively. The solid samples were then dried under vacuum at 60 ˚C for 4 h. The obtained photocatalyst was labelled as H-CdS.

Preparation of Cubic CdS

308 mg $Cd(NO_3)_2·4H_2O$ and 1.2 g $Na_2S·9H_2O$ were dissolved into 15 ml of water. After stirring for 1 h, the orange slurry solution was formed. The solid sample was collected by centrifugation (9000 rpm), rinsed several times with ethanol and water respectively. The solid sample was then dried under vacuum at 60 ˚C for 4 h. The obtained photocatalyst was labelled as C-CdS.

Scherrer Equation for Crystal Sizes of Both Hexagonal and Cubic Phases in CdS-n Photocatalysts

The average crystal sizes of both the hexagonal and cubic phases in CdS-n photocatalysts are calculated from the XRD spectra using the Scherrer equation:

$$D = \frac{\kappa\lambda}{\beta cos\theta}$$

Where κ is the Scherrer constant (κ = 0.934), λ is the wavelength of the X-ray radiation (λ = 0.154056 nm), θ is X-ray diffraction angle and β is the full width at half-maximum (FWHM) of crystal facets. The identified crystal facets for the hexagonal phase include (100), (002), (101), (102), (110), (103), and (112), while the cubic phase contains (111), (200), (220), and (311) facets.

The Stern-Volmer equation

The Stern-Volmer quenching constants of PP-ol, PP-one and TEA were calculated using the Stern-Volmer equation:

$$K_{SV}[Q] = \frac{I_0}{I} - 1$$

Where, I_0 and I are the photoluminescence emission intensities in the absence and presence of each quencher, such as PP-ol, PP-one and TEA, K_{SV} is the Stern–Volmer constant, [Q] is the concentration of each quencher. The quenchers include PP-ol, PP-one and TEA.

Texture Coefficient Calculation.

The texture coefficient of hexagonal (100) and cubic (220) facets are calculated from the XRD spectrum according to the following formula:

$$TC_{(h_ik_il_i)} = \frac{\frac{I_{(h_ik_il_i)}}{I_{0(h_ik_il_i)}}}{\frac{1}{n}\{\sum_{i=1}^{n}\frac{I_{(h_ik_il_i)}}{I_{0(h_ik_il_i)}}\}}$$

Where $I_{(h_ik_il_i)}$ is the intensity of $(h_ik_il_i)$ diffraction peak on the phase junction CdS under investigation; $I_{0(h_ik_il_i)}$ is the intensity of $(h_ik_il_i)$ plane of the standard profile from a powder diffraction file (PDF) card; and n is the number of diffractions considered in the analysis. For the hexagonal (100) facet: n = 7; cubic (220) facet: n = 4.

DFT calculation

The first-principal calculations based on density functional theory (DFT) were carried out using the Materials Studio 2020 software (Biovia, San Diego). The density of states and work functions were determined using the Cambridge Serial Total Energy Package (CASTEP) module. The generalized gradient approximation-Perdew Burke Ernzerhof (GGA-PBE) was employed as the exchange–correlation functional. An adequate vacuum space of 15 Å was applied. The cut-off energies and k-points were set to 500 eV and 2 × 2 × 1, respectively.

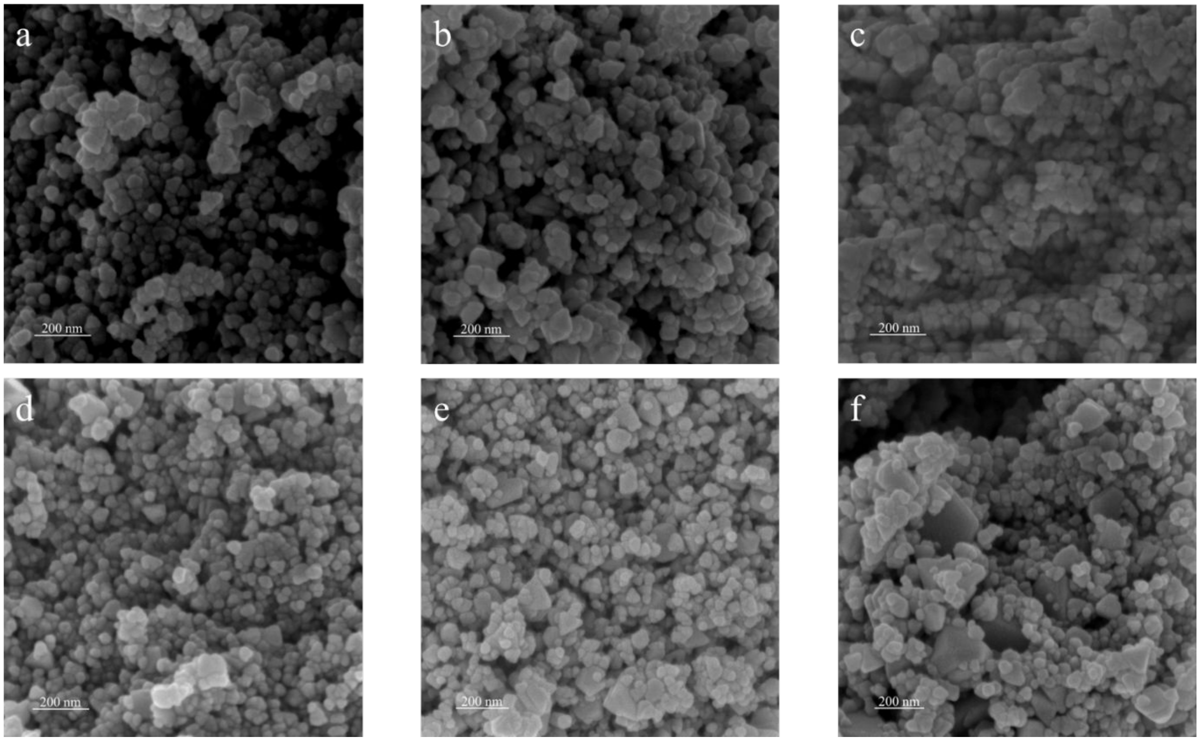

Figure S1. SEM images of (a) CdS-0, (b) CdS-50, (c) CdS-100, (d) CdS-150, (e) CdS-200, (f) CdS-300.

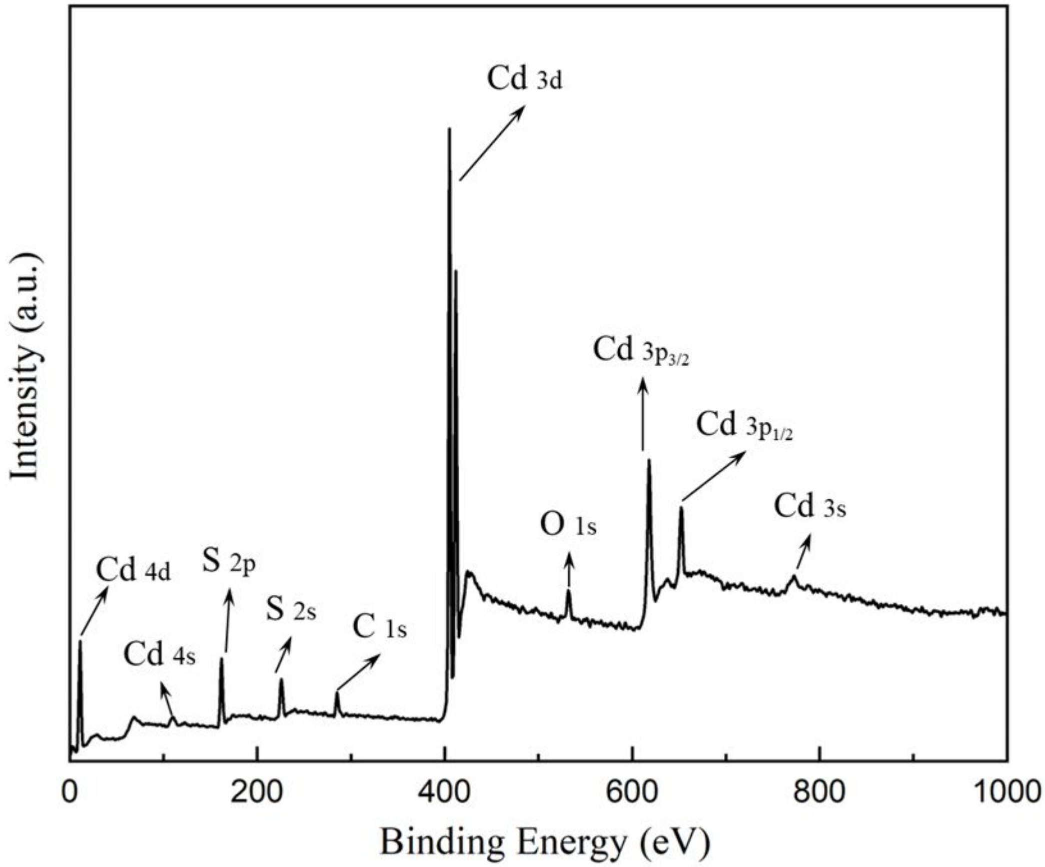

Figure S2. Full XPS spectrum of CdS-150.

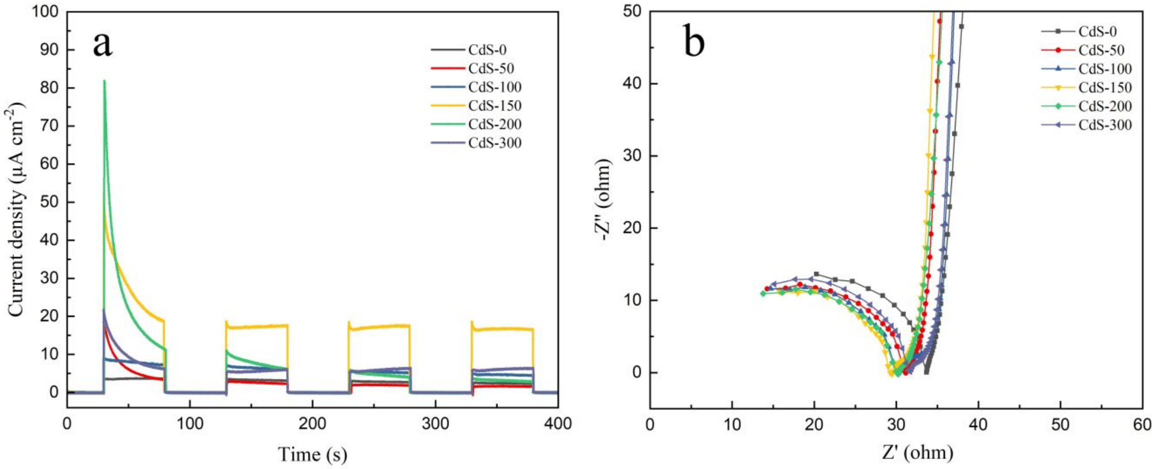

Figure S3. PEC properties for CdS-n in the presence of PP-ol. (a) Transient photocurrent responses. (b) EIS spectra.

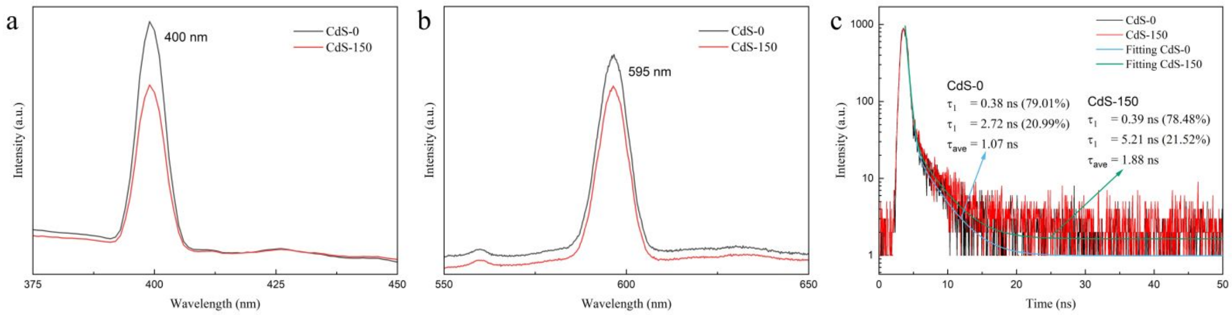

Figure S4. (a) Excitation spectra ($\lambda_{emission}$ = 595 nm), and (b) emission spectra ($\lambda_{excitation}$ = 400 nm) of CdS-0 and CdS-150 using powdery photoluminescence (PL) method. (c) Time-resolved transient photoluminescence (TRPL) spectra of CdS-0 and CdS-150.

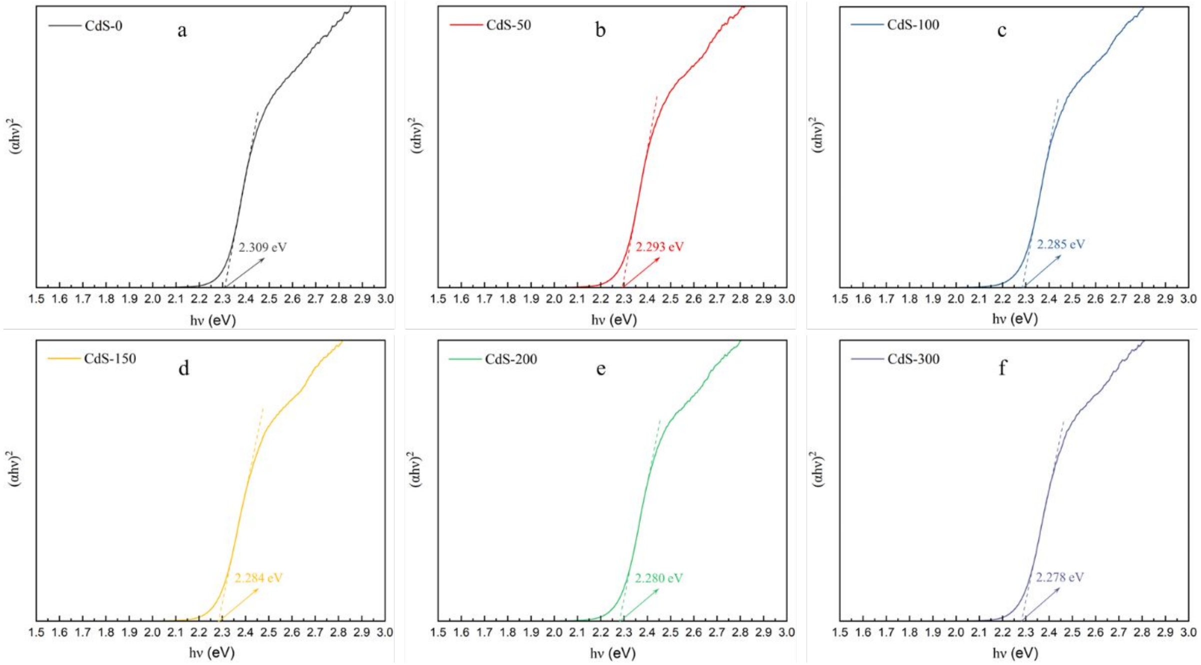

Figure S5. $(\alpha h\nu)^2$ plots versus hν curves for (a) CdS-0, (b) CdS-50, (c) CdS-100, (d) CdS-150, (e) CdS-200, (f) CdS-300.

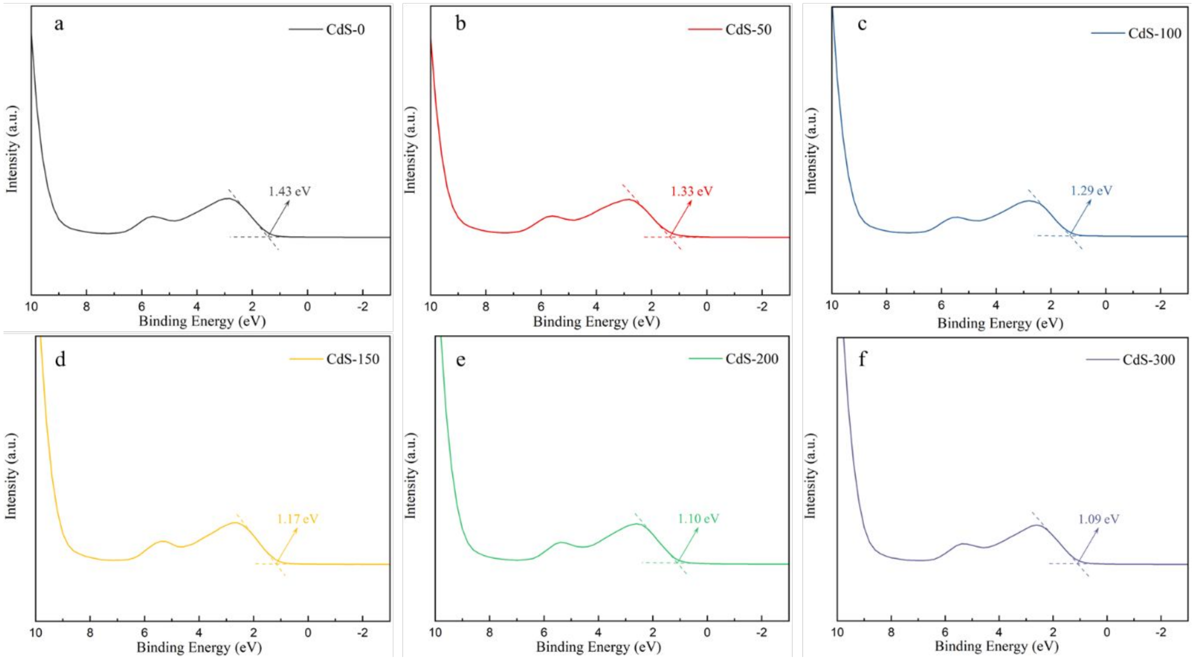

Figure S6. Valence-band XPS spectra for (a) CdS-0, (b) CdS-50, (c) CdS-100, (d) CdS-150, (e) CdS-200, (f) CdS-300.

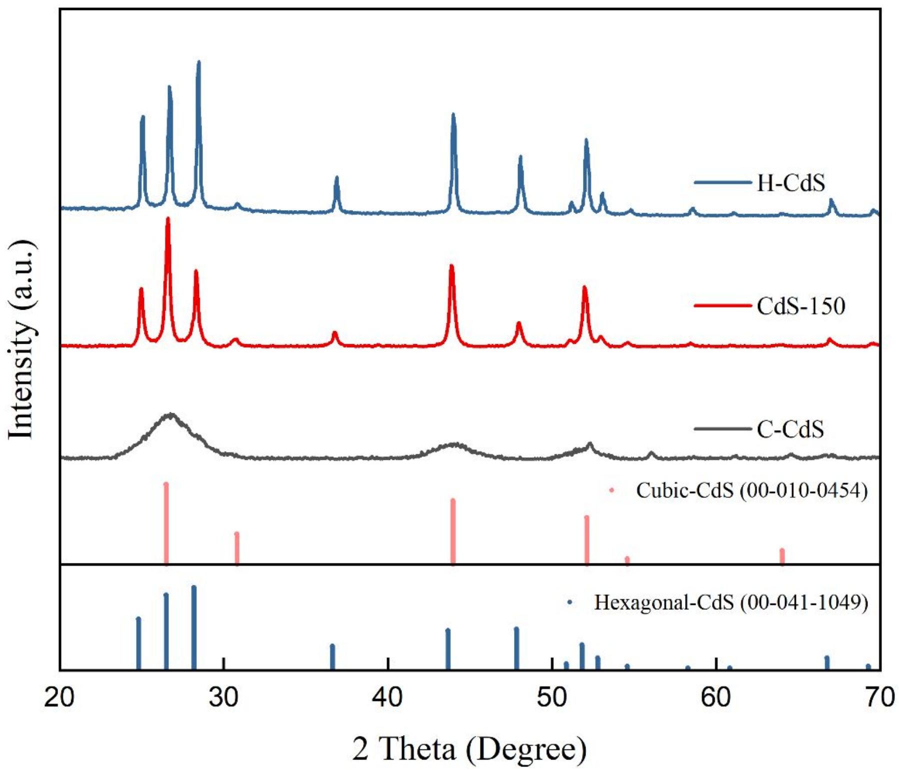

Figure S7. XRD patterns of cubic CdS (C-CdS), phase junction CdS (CdS-150), hexagonal CdS (H-CdS).

Table S1. Photocatalytic performance of heterogenous catalysts for PP-ol conversion in literature

Ref	Catalyst	Reaction Condition						Conversion	Selectivity		
		Solvent	PP-ol amount	Temp	Atmosphere	Reaction time	Light Source	PP-ol	Phenol	Acetophenone	PP-one
1	$ZnIn_2S_4$ (5 mg)	1 mL CH_3CN	0.10 mmol	42 °C	N_2	4 h	9.6 W blue LEDs (455 nm),	>99%	90%	83%	6%
2	$Zn_4In_2S_7$ (10 mg)	5 mL CH_3CN/H_2O (1:1 v/v)	0.10 mmol	-	N_2	4 h	Xe lamp (400–780 nm), 0.6 W/cm^2	99%	82%	86%	9.6%
3	Ni/CdS (20 mg)	10 mL CH_3CN/0.1 M KOH (2:8, v/v)	0.10 mmol	-	N_2	2 h	8 W Blue LED (440–460 nm)	~100%	~90%	~90%	-
4	Ag2S@ CdS (1 mg)	1 mL CH_3CN	10 mg	30 °C	Ar	3 h	6 W Blue LED	99%	95%	91%	4%
5	ZIS-3 (10 mg)	5 mL CH_3CN/H_2O (2:3, v/v)	10 mg	20-25 °C	Ar	1.5h	Xe lamp, (420–780 nm, $0.35W/cm^2$)	~100%	93.7%	91.9%	4.7%
6	SL-Fe_3O_4/TiO_2 (100mg)	50 mL methanol, 0.5 mL 30% H_2O_2	0.5 mmol	40 °C	O_2	12 h	-	94.3%	3.0%	3.5%	-
7	ZIF-8-NH_2@Bi/Bi_2MoO_6 (10 mg)	10 mL CH_3CN/H_2O (1:1, v/v)	10 mL of PP-ol (0.05 mmol/L)	20-25 °C	Air	6h	300 W xenon lamp ($\lambda > 400$ nm)	93%	57%	48%	34%
8	CdS−SH/TiO_2 (10 mg)	5 mL CH_3CN	1 mg	20 °C	N_2	1h	300 W xenon lamp	99%	87%	85%	<1%
This work	CdS-150 (10mg)	5 mL CH_3CN/H_2O (2:3, v/v)	10 mg	20 °C	He	1h	Xe lamp, (420–780 nm, $0.35W/cm^2$)	100%	94.3%	93.4%	3.6%

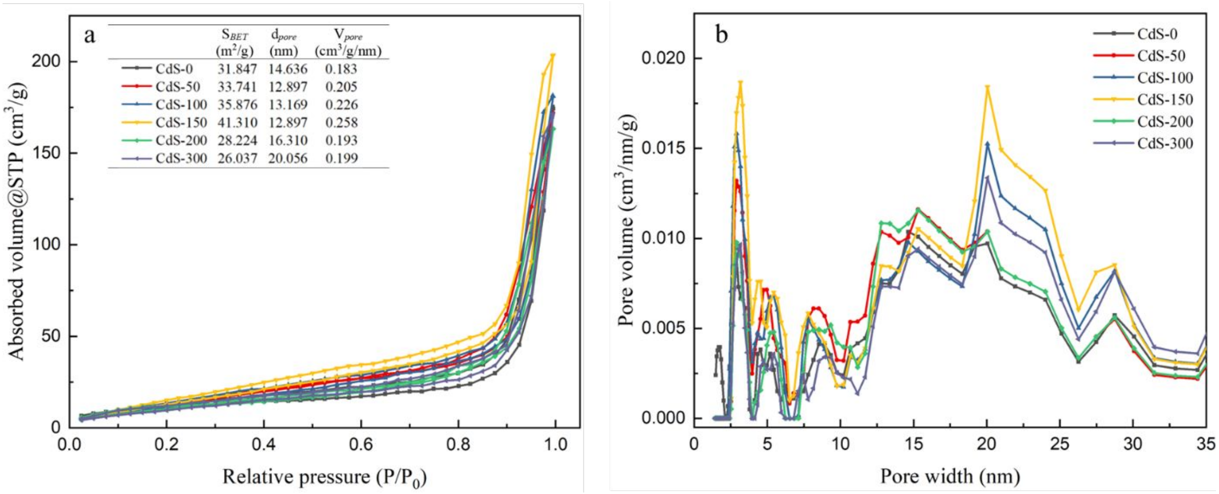

Figure S8. (a) Nitrogen adsorption/desorption isotherms and (b) pore size distribution curves of CdS. The insert table in (a) includes the surface area, pore diameter and pore volume.

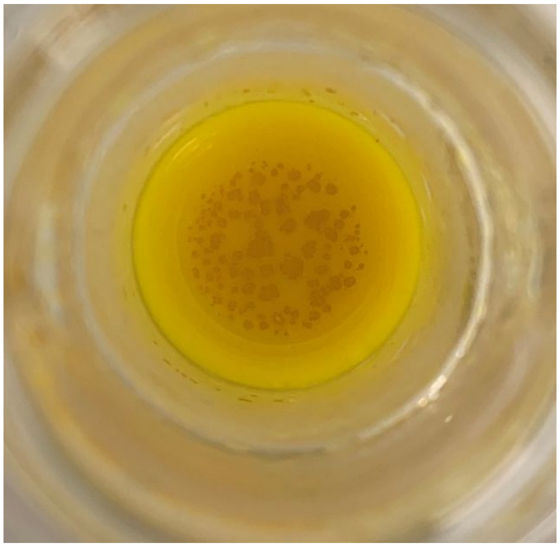

Figure S9. The image of the reaction solution after 1h photocatalytic reaction. Reaction condition: lignin model compound PP-ol is 10 mg, CdS-150 is 10 mg, solvent (CH_3CN/H_2O (v/v = 2/8)) is 5 ml, Ar is at 1 atm, visible light (0.35 W cm^{-2}), 1 h.

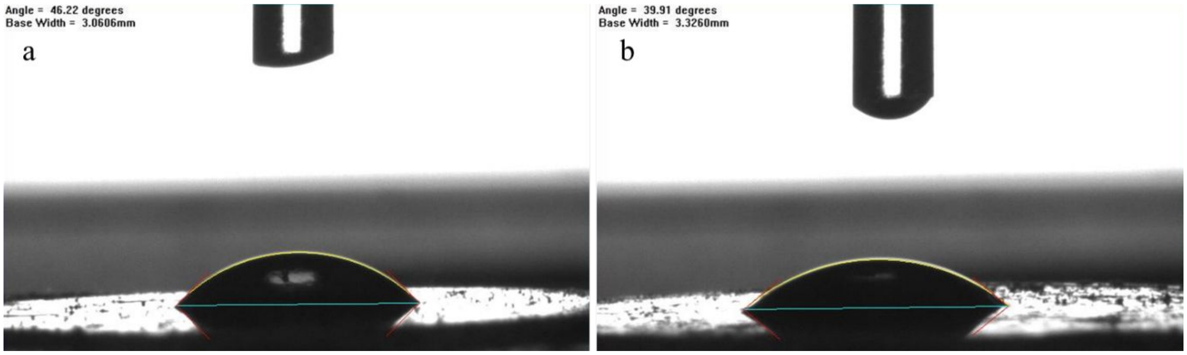

Figure S10. Contact angle of (a) CdS-0 and (b) CdS-150.

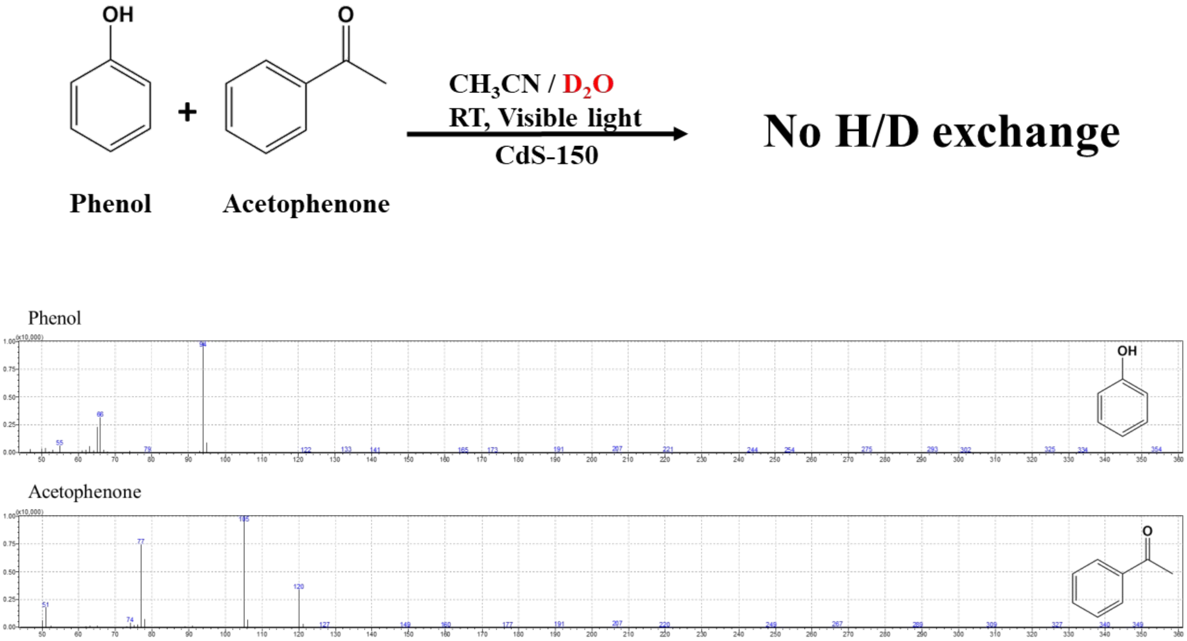

Figure S11. Phenol and acetophenone in D_2O reaction system for H/D exchange. Reaction condition: phenol is 10 mg, acetophenone is 10 mg, CdS-150 is 10 mg, solvent (CH_3CN/D_2O (v/v = 3/7)) is 5 ml, Ar is at 1 atm, visible light power is 0.35 W cm^{-2}, 30 min.

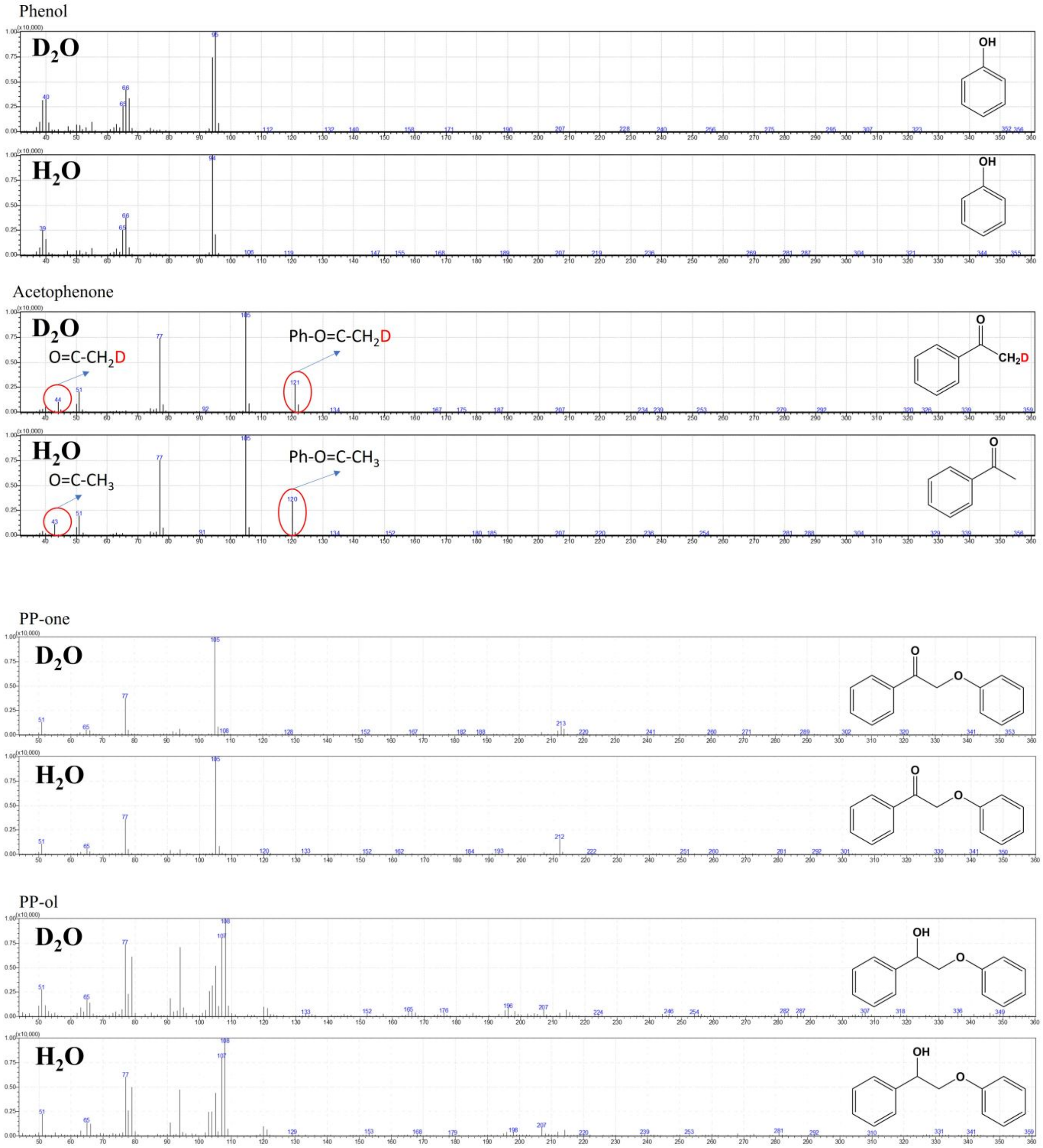

Figure S12. The mass spectra of the fragmented PP-ol to acetophenone, phenol and PP-one in H_2O and D_2O solvent conditions. Reaction condition: lignin model compound PP-ol is 10 mg, CdS-150 is 10 mg, solvent ($CH_3CN/H_2O(D_2O)$ (v/v = 3/7)) is 5 ml, Ar is at 1 atm, visible light power is 0.35 W cm^{-2}, 30 min.

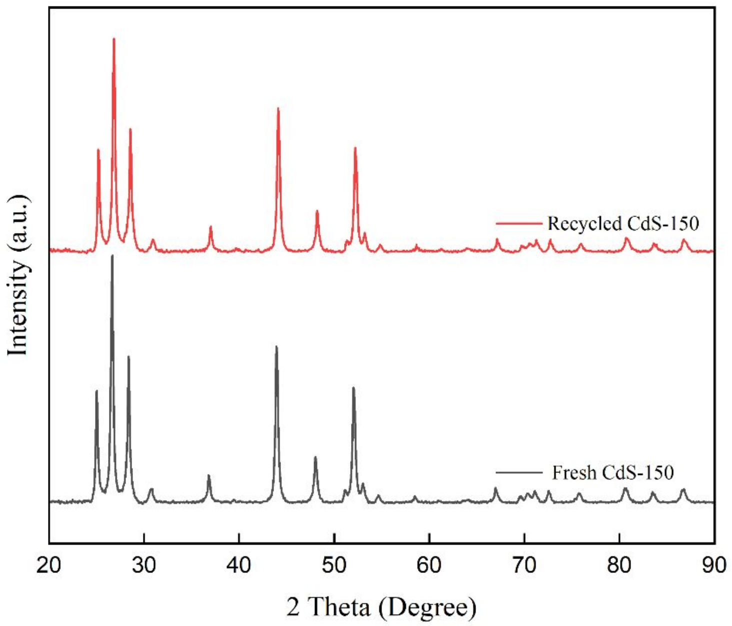

Figure S13. XRD patterns of fresh and recycled CdS-150.

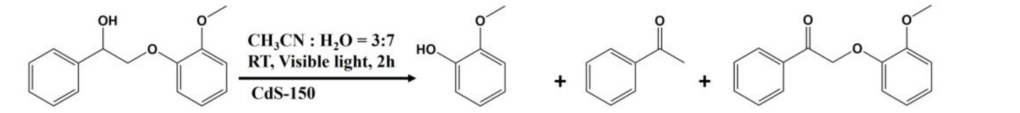

2-(3-methoxyphenoxy)-1-phenylethanol (MP-ol) (100% conversion)

Guaiacol (90.1% yield)

Acetophenone (97.8% yield)

2-(4-methoxyphenoxy)-1-phenyl-ethenone (MP-one) (2.5% yield)

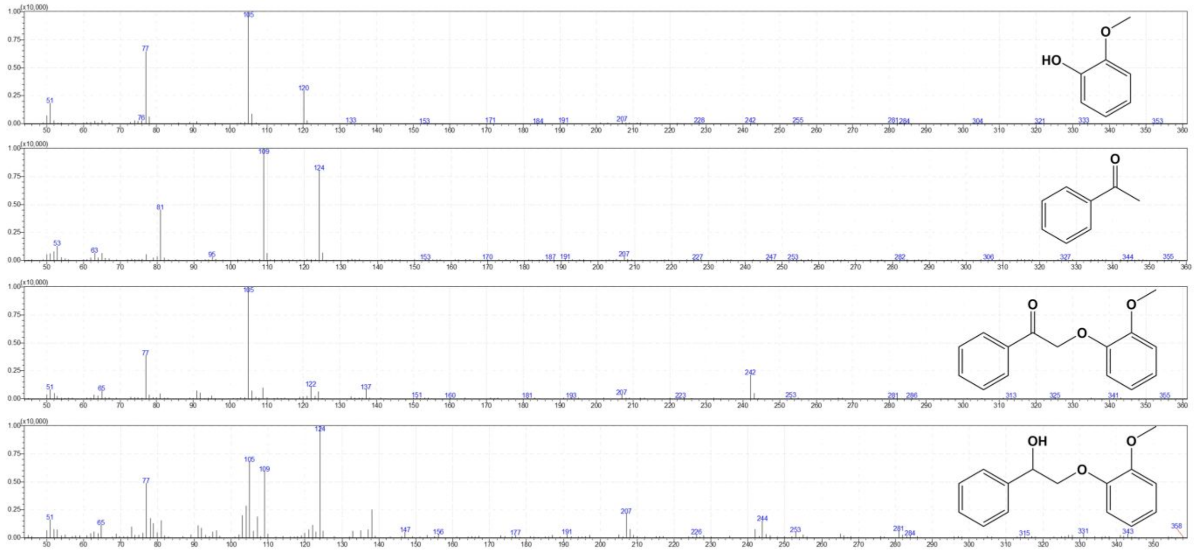

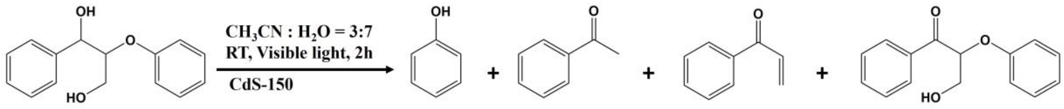

2-Phenoxy-1-phenylpropane-1,3-diol (PPP-ol) (100% conversion)

Phenol (60.6% yield)

Acetophenone (28.6% yield)

Acrylophenone (53.2% yield)

2-phenoxy-1-phenylpropan-1-one (PPP-one) (15.7% yield)

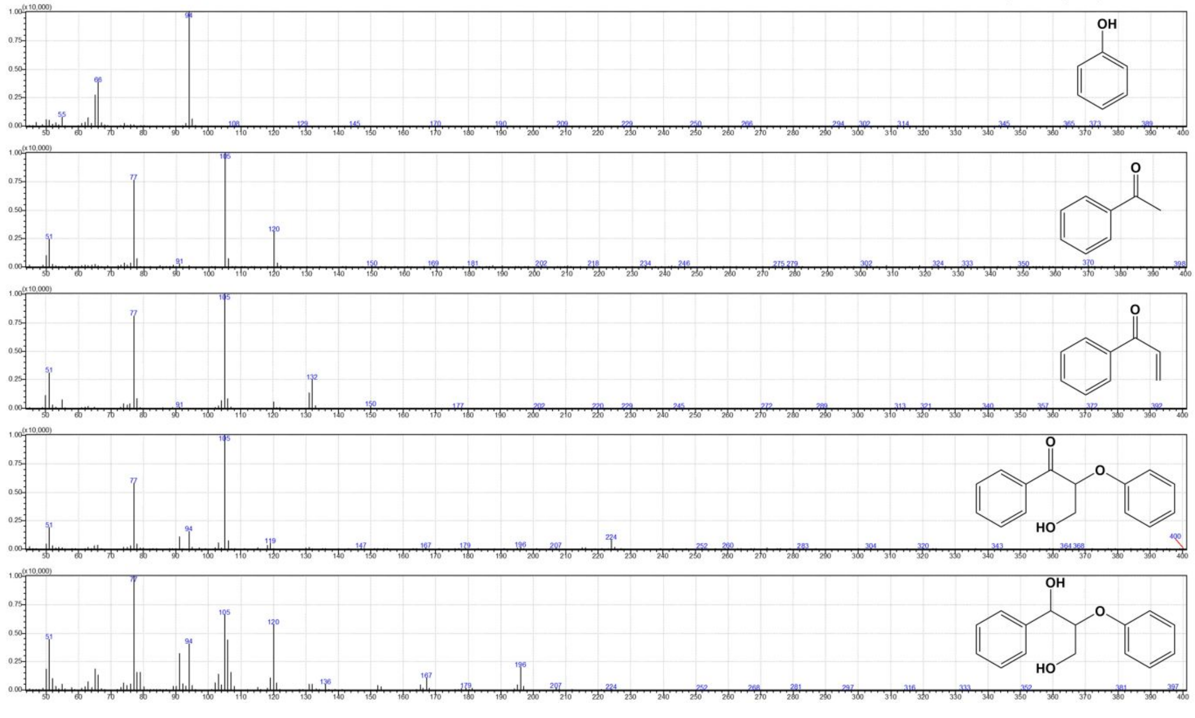

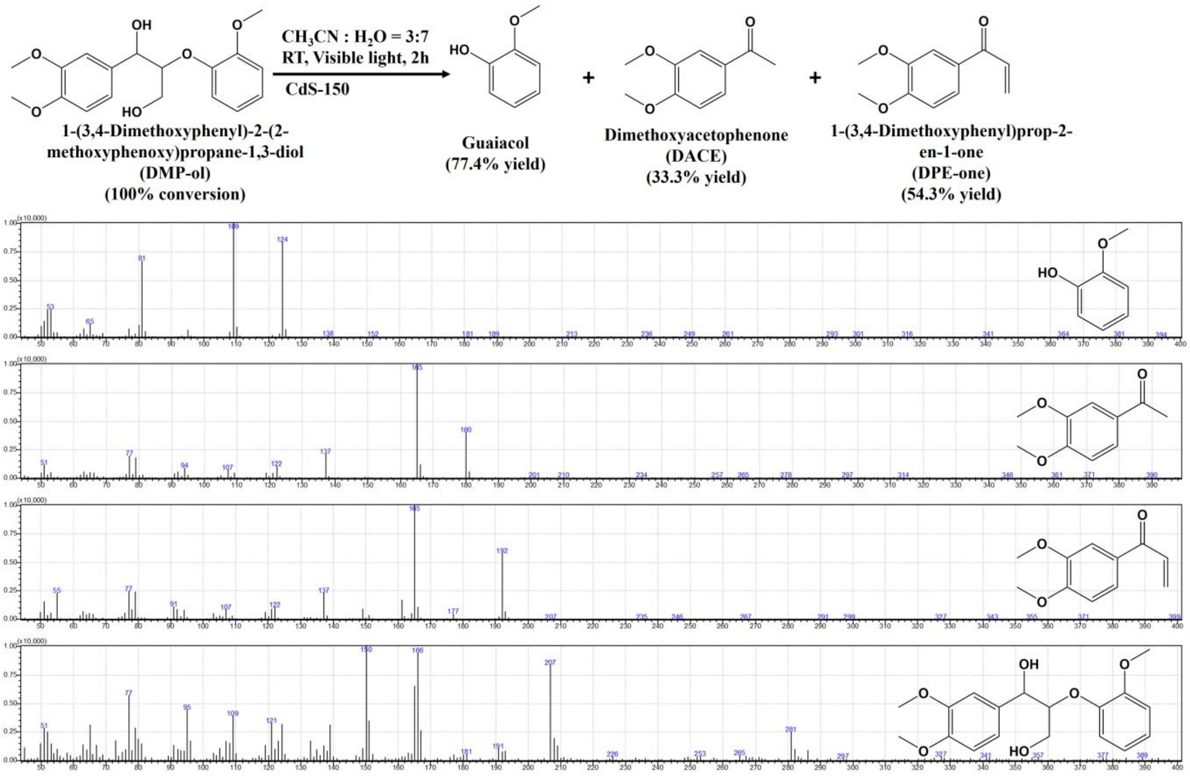

Figure S14. The mass spectra of the fragmented lignin models to desirable aromatic monomers. Reaction condition: lignin model compound is 10 mg, CdS-150 is 10 mg, solvent (CH_3CN/H_2O (v/v = 3/7)) is 5 ml, Ar is at 1 atm, visible light power is 0.35 W cm^{-2}, 2 h.

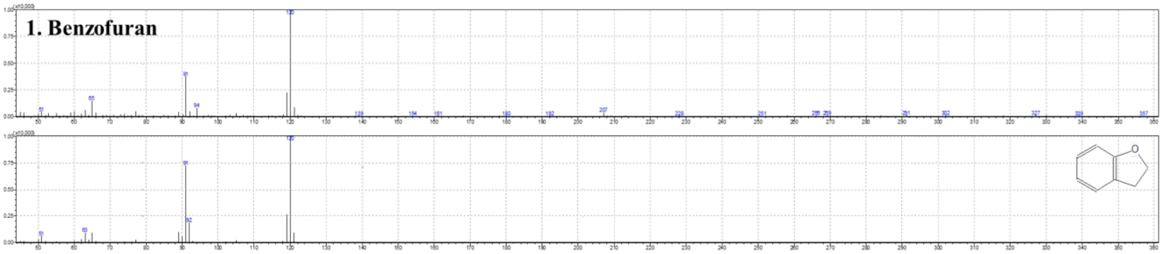
1. Benzofuran

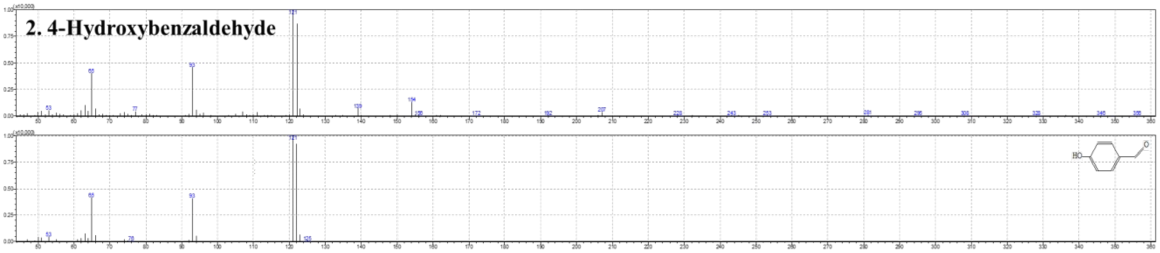
2. 4-Hydroxybenzaldehyde

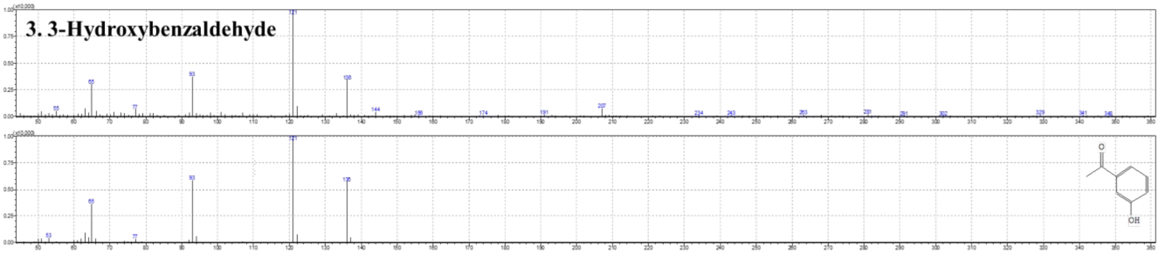
3. 3-Hydroxybenzaldehyde

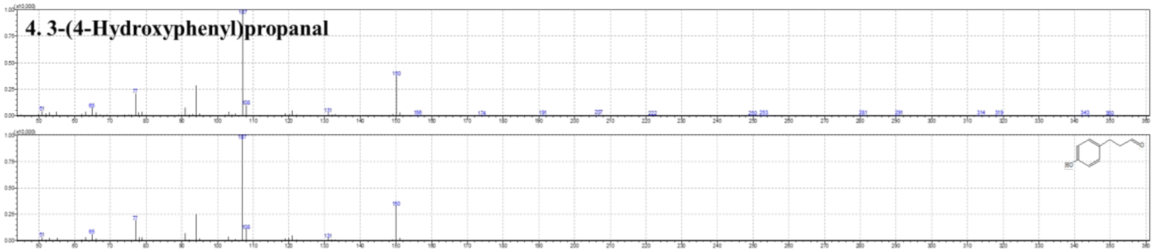
4. 3-(4-Hydroxyphenyl)propanal

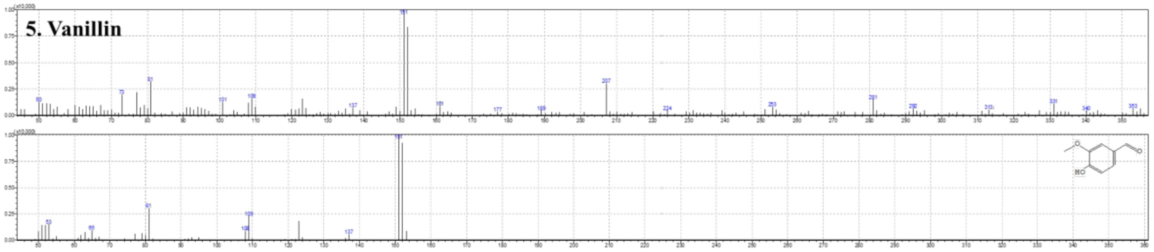
5. Vanillin

6. 4-(4-Hydroxyphenyl)-2-butanone

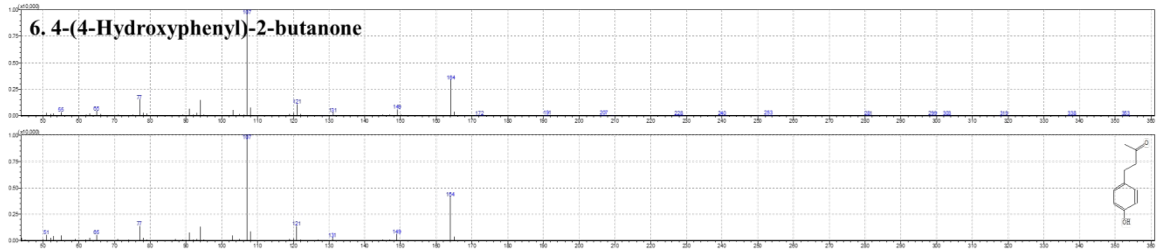

7. 4-(3-Hydroxybutyl)phenol

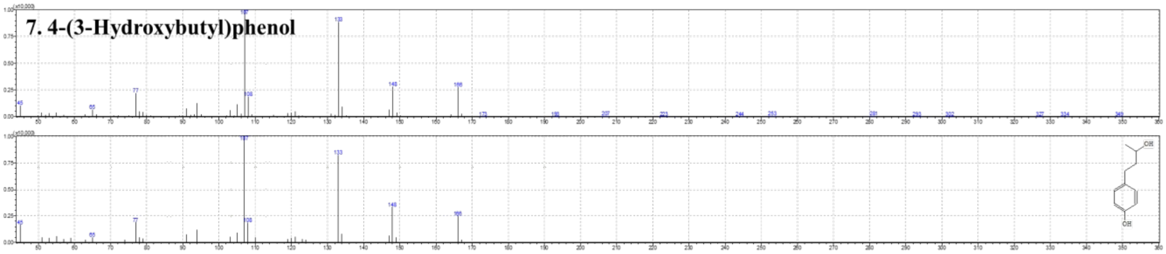

8. 3,4,5-Trimethoxyphenol

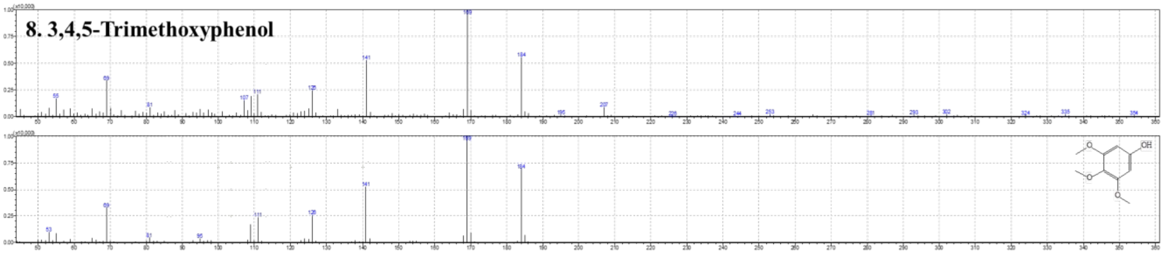

9. Phenacyl acetate

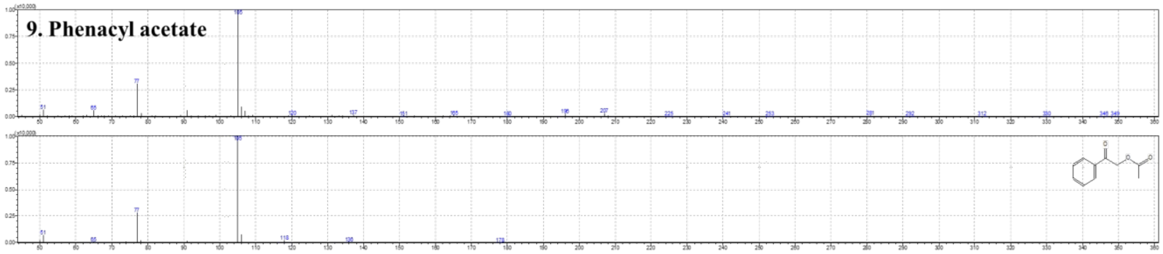

10. 2-(3,4,5-trihydroxyphenyl)acetic Acid

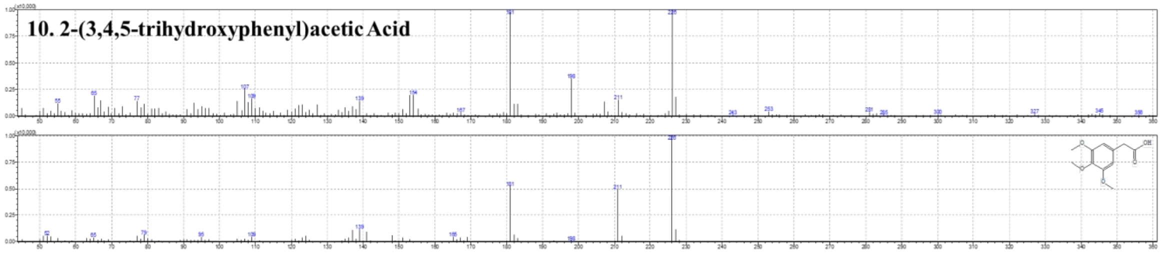

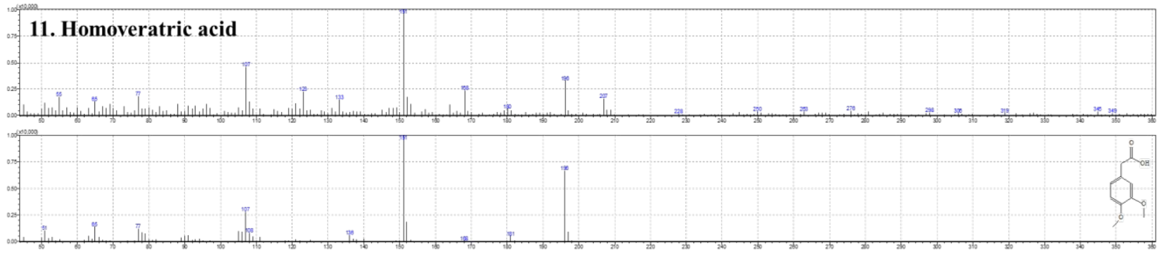

Figure S15. The detected and the standard mass spectra of different aromatic monomers from fragmentation of wood extraction lignin in 10h of visible light irradiation. Reaction conditions: wood extraction powders are 80 mg, CdS-150 is 20 mg, H_2O and CH_3CN mixed solution (CH_3CN/H_2O (v/v = 3/7)) is 7 ml, Ar is at 1 atm, visible light power is 0.35 W cm^{-2}, 10 h.

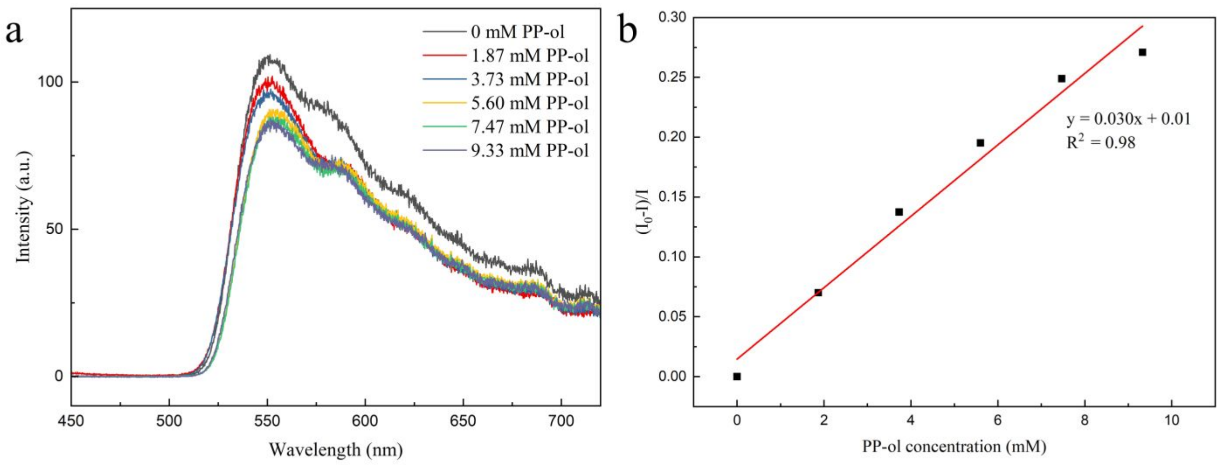

Figure S16. (a) Photoluminescence emission spectra of CdS with varying concentration of PP-ol under visible light irradiation (excitation wavelength at 420 nm). (b) The calculated Stern–Volmer quenching constant of CdS-150 in presence of PP-ol ($K_{SV(PP\text{-}ol)}$).

Table S2. Photocatalytic performance of heterogenous catalysts for ketone compounds conversion in literature

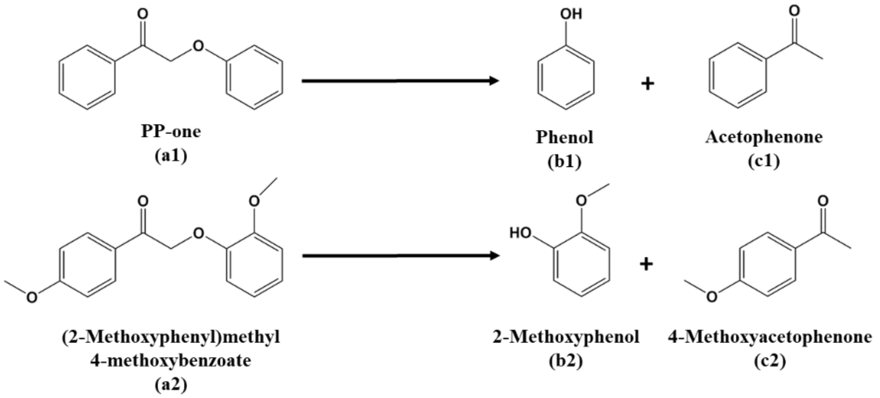

Ref	Catalyst	Reaction Condition						Conversion	Selectivity	
		Solvent	Reactant	Temp	Atmosphere	Reaction time	Light Source	a1 / a2	b1 / b2	c1 / c2
9	TiO_2 (5 mg)	0.5 equiv NaOAc, 0.75ml ethanol	a1, 0.10 mmol	65 ˚C	N_2	5 h	5.6 W blue LEDs (365 nm),	>99%	93%	90%
10	$ZnIn_2S_4$ (10 mg)	4 mL CH_3CN, 1 ml isopropanol	a1, 0.10 mmol	17-23 ˚C	N_2	2 h	300 W Xe-lamp	69.7%	65.3%	60%
11	Ni/TiO_2 (20 mg)	2.5 ml DMF	a1, 100 mg	RT	Air	12 h	30 W UV	100%	82%	83%
12	[Ir(ppy)$_2$(dtbbpy)]PF_6 (1.0 mol %)	5 ml methanol, 3.0 equiv DIPEA, 3.0 equiv, HCO_2H	a2, 1.0 mmol	RT	-	12 h	7 blue LEDs (λ_{max} = 447.5 nm)	100%	89%	88%
13	CzCP33 (2.0 mol%)	4 ml methanol, 1.0mmol DIPEA, 1.0 mmol HCOOH	a2, 0.2 mmol	-	Ar	24 h	26 W CFL	100%	86%	89%
This work	CdS-150 (10mg)	5 mL CH_3CN:H_2O (2:3, v/v), 0.5 ml TEA	10 mg	20 ˚C	He	10 min	Xe lamp, (420–780 nm, 0.35W/cm^2)	100%	95.1%	98.2%

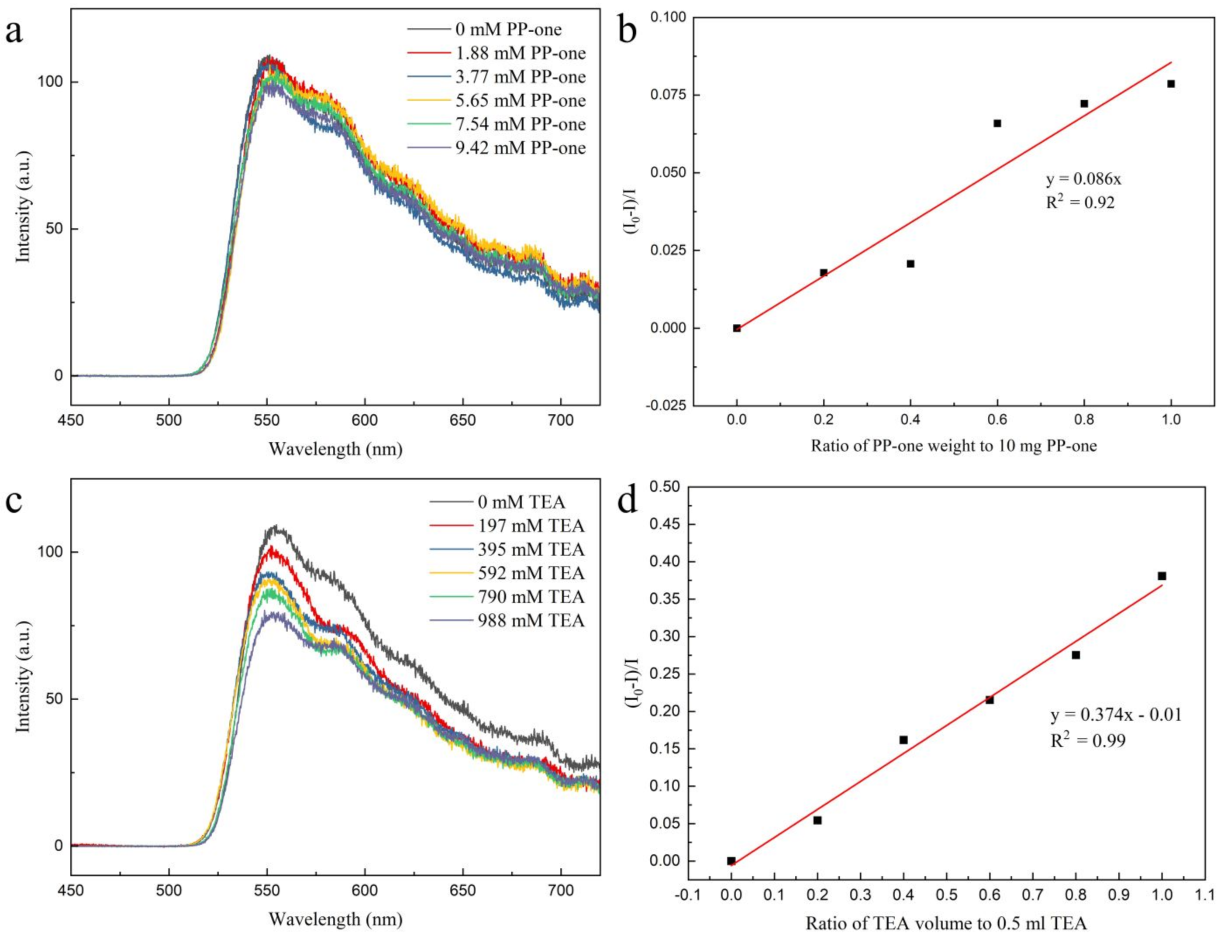

Figure S17. (a) Photoluminescence emission spectra of CdS with varying concentration of PP-one under visible light irradiation (excitation wavelength at 420 nm). (b) The calculated Stern–Volmer quenching constant of CdS in presence of PP-one ($K_{SV(PP\text{-}one)}$). (c) Photoluminescence emission spectra of CdS with varying concentration of TEA under visible light irradiation (excitation wavelength at 420 nm). (d) The calculated Stern–Volmer quenching constant of CdS in presence of TEA ($K_{SV(TEA)}$).

Note:

To calculate the K_{SV} of TEA and PP-one, the dimensionless ratios of TEA and PP-one were conducted to replace their actual concentrations. As the concentration of 0.5 ml TEA in 5 ml reaction solvent is 988 mM which is much higher than that of 10 mg PP-one (9.42 mM), the calculated K_{SV} using their actual concentrations is 0.009 for $K_{SV(PP\text{-}one)}$ and 0.0004 for $K_{SV(TEA)}$, which is difficult to reflect the actual role of each quencher in hole transfer from CdS to PP-one. Hence, 10 mg PP-one and 0.5 ml TEA in 5 ml reaction solvent were set as the baseline values. The dimensionless ratio of TEA is the ratio of varying TEA volumes to 0.5 ml TEA in 5 ml reaction solvent, and the dimensionless ratio for PP-one is the ratio of varying PP-one weight to 10 mg PP-one in 5 ml reaction solvent. The calculated K_{SV} in this way provides a better comparison in the contributions of PP-one and TEA for quenching experiments.

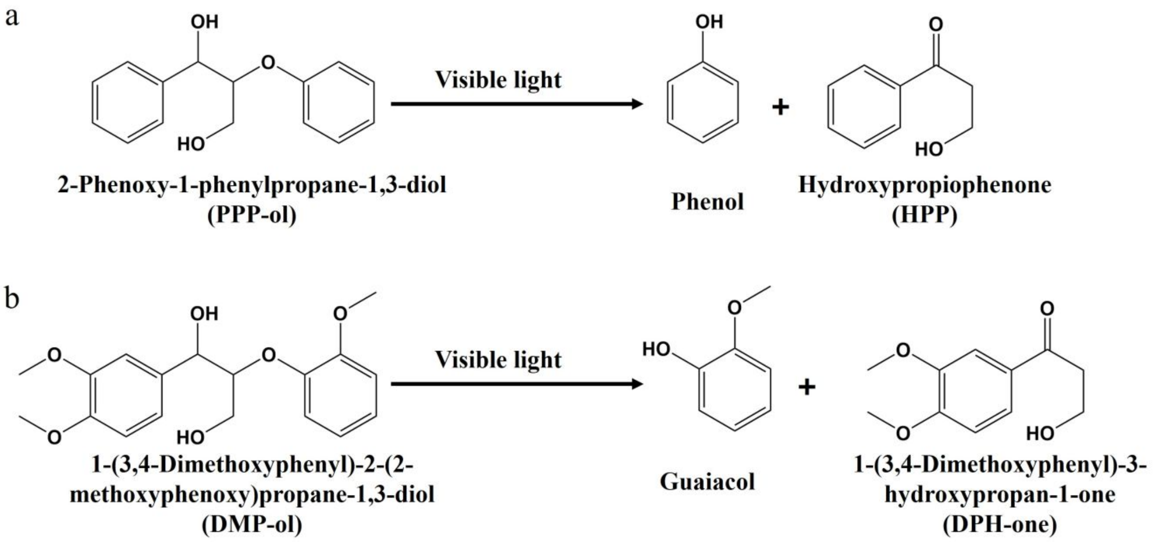

Scheme S1. Conversion of (a) PPP-ol and (b) DMP-ol to phenol-like products and acetophenone-like products from previous works.[6,12,13,16,17]

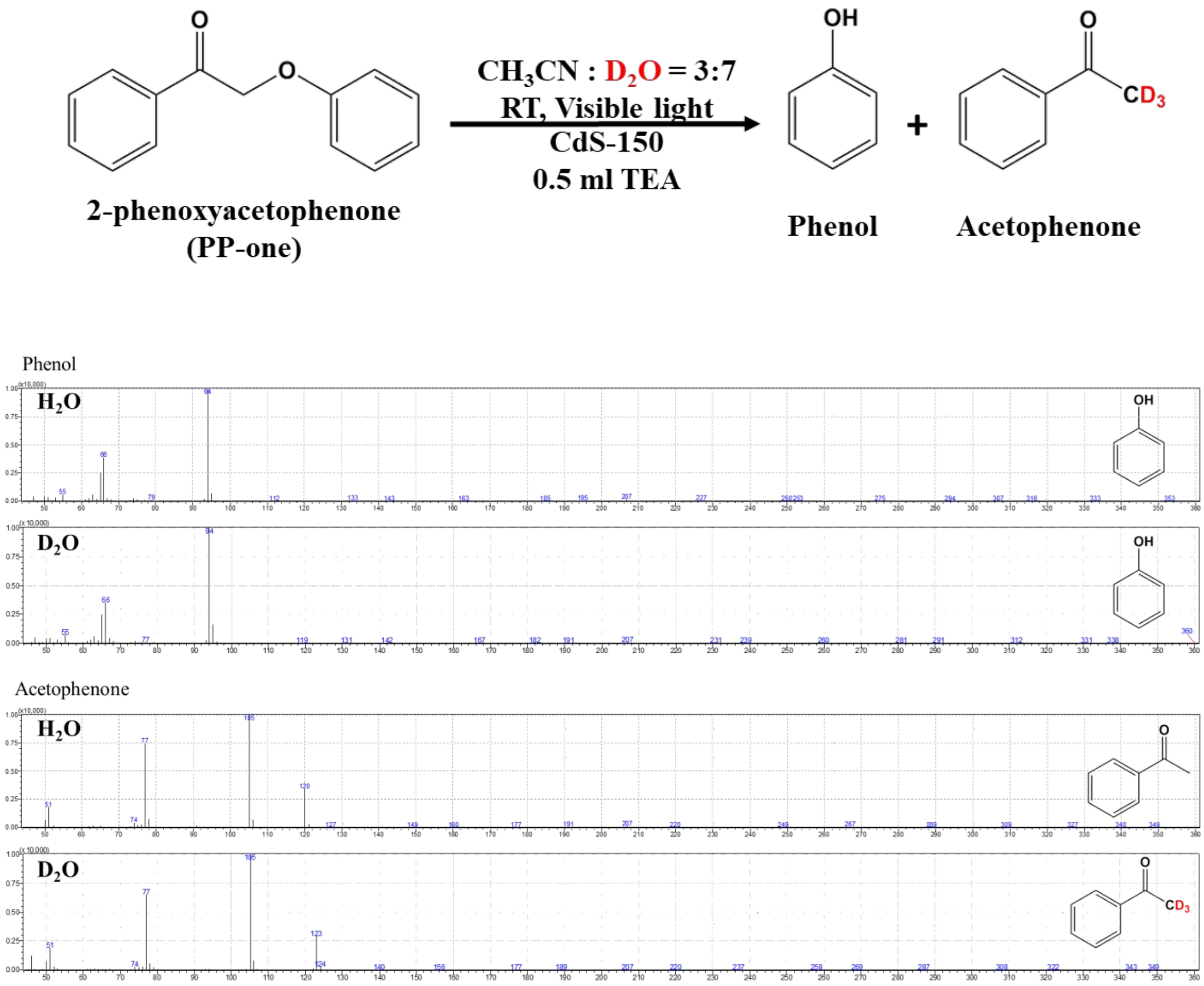

Fig, S18. The detected mass spectra of the fragmented PP-one to acetophenone and phenol in D_2O system. Reaction condition: PP-one is 10 mg, photocatalyst is 10 mg, solvent (CH_3CN/D_2O (v/v = 3/7)) is 4.5 ml, TEA is 0.5 ml, Ar is at 1 atm, visible light power is 0.35 W cm^{-2}, 10 min.

Reference

(1) Luo, N.; Wang, M.; Li, H.; Zhang, J.; Hou, T.; Chen, H.; Zhang, X.; Lu, J.; Wang, F. Visible-Light-Driven Self-Hydrogen Transfer Hydrogenolysis of Lignin Models and Extracts into Phenolic Products. *ACS Catal.* **2017**, *7* (7), 4571–4580. https://doi.org/10.1021/acscatal.7b01043.

(2) Lin, J.; Wu, X.; Xie, S.; Chen, L.; Zhang, Q.; Deng, W.; Wang, Y. Visible-Light-Driven Cleavage of C−O Linkage for Lignin Valorization to Functionalized Aromatics. *ChemSusChem* **2019**, *12* (22), 5023–5031. https://doi.org/10.1002/cssc.201902355.

(3) Han, G.; Yan, T.; Zhang, W.; Zhang, Y. C.; Lee, D. Y.; Cao, Z.; Sun, Y. Highly Selective Photocatalytic Valorization of Lignin Model Compounds Using Ultrathin Metal/CdS. *ACS Catal.* **2019**, *9* (12), 11341–11349. https://doi.org/10.1021/acscatal.9b02842.

(4) Yoo, H.; Lee, M. W.; Lee, S.; Lee, J.; Cho, S.; Lee, H.; Cha, H. G.; Kim, H. S. Enhancing Photocatalytic β-O-4 Bond Cleavage in Lignin Model Compounds by Silver-Exchanged Cadmium Sulfide. *ACS Catal.* **2020**, *10* (15), 8465–8475. https://doi.org/10.1021/acscatal.0c01915.

(5) Shao, S.; Wang, K.; Love, J. B.; Yu, J.; Du, S.; Yue, Z.; Fan, X. Water Promoted Photocatalytic $C_β$-O Bonds Hydrogenolysis in Lignin Model Compounds and Lignin Biomass Conversion to Aromatic Monomers. *Chem. Eng. J.* **2022**, *435* (P2), 134980.

https://doi.org/10.1016/j.cej.2022.134980.

(6) Wu, K.; Liang, J.; Liu, S.; Huang, Y.; Cao, M.; Zeng, Q.; Li, X. Selective Photocatalytic Aerobic Oxidative Cleavage of Lignin C–O Bonds over Sodium Lignosulfonate Modified Fe3O4/TiO2. *J. Energy Chem.* **2023**, *84*, 89–100. https://doi.org/10.1016/j.jechem.2023.04.033.

(7) Dai, D.; Qiu, J.; Xia, G.; Tang, Y.; Yao, J. Defect Engineering Promoted Photocatalysis for Lignin Depolymerization: Performance and Mechanism Insight. *ACS Catal.* **2023**, *13* (22), 14987–14995. https://doi.org/10.1021/acscatal.3c03462.

(8) Xu, S.; Gao, Q.; Hu, Z.; Lu, Y.; Qin, Y.; Li, Y. CdS–SH/TiO_2 Heterojunction Photocatalyst Significantly Improves Selectivity for C–O Bond Breaking in Lignin Models. *ACS Catal.* **2023**, *13* (21), 13941–13954. https://doi.org/10.1021/acscatal.3c03309.

(9) Luo, N.; Wang, M.; Li, H.; Zhang, J.; Liu, H.; Wang, F. Photocatalytic Oxidation-Hydrogenolysis of Lignin β-O-4 Models via a Dual Light Wavelength Switching Strategy. *ACS Catal.* **2016**, *6* (11), 7716–7721. https://doi.org/10.1021/acscatal.6b02212.

(10) Xu, J.; Li, M.; Qiu, J.; Zhang, X. F.; Yao, J. Photocatalytic Depolymerization of Organosolv Lignin into Valuable Chemicals. *Int. J. Biol. Macromol.* **2021**, *180*, 403–410. https://doi.org/10.1016/j.ijbiomac.2021.03.067.

(11) Chen, C.; Liu, P.; Xia, H.; Zhou, M.; Zhao, J.; Sharma, B. K.; Jiang, J. Photocatalytic

Cleavage of β-O-4 Ether Bonds in Lignin over Ni/TiO2. *Molecules* **2020**, *25* (9), 1–14. https://doi.org/10.3390/molecules25092109.

(12) Nguyen, J. D.; Matsuura, B. S.; Stephenson, C. R. J. A Photochemical Strategy for Lignin Degradation at Room Temperature. *J. Am. Chem. Soc.* **2014**, *136* (4), 1218–1221. https://doi.org/10.1021/ja4113462.

(13) Luo, J.; Zhang, X.; Lu, J.; Zhang, J. Fine Tuning the Redox Potentials of Carbazolic Porous Organic Frameworks for Visible-Light Photoredox Catalytic Degradation of Lignin β-O-4 Models. *ACS Catal.* **2017**, *7* (8), 5062–5070. https://doi.org/10.1021/acscatal.7b01010.
